# Socioeconomic status and health behavior in children and adolescents: a systematic literature review

**DOI:** 10.3389/fpubh.2023.1228632

**Published:** 2023-10-17

**Authors:** Nirmal Gautam, Getenet Dessie, Mohammad Mafizur Rahman, Rasheda Khanam

**Affiliations:** ^1^School of Business, Faculty of Business, Education, Law and Arts, University of Southern Queensland, Toowoomba, QLD, Australia; ^2^Centre for Health Research, University of Southern Queensland, Toowoomba, QLD, Australia; ^3^National Centre for Epidemiology and Population Health, College of Health and Medicine, Australian National University, Canberra, ACT, Australia; ^4^College of Medicine and Health Sciences, Bahir Dar University, Bahir Dar, Ethiopia

**Keywords:** socioeconomic status, health behavior, childhood, adolescence, systematic literature review

## Abstract

Socioeconomic status affects individuals’ health behaviors and contributes to a complex relationship between health and development. Due to this complexity, the relationship between SES and health behaviors is not yet fully understood. This literature review, therefore, aims to assess the association between socioeconomic status and health behaviors in childhood and adolescence. Preferred Reporting for Systematic Review and Meta-Analysis protocol guidelines were used to conduct a systematic literature review. The electronic online databases EBSCO Host, PubMed, Web of Science, and Science Direct were utilized to systematically search published articles. The Joanna Briggs Institute’s critical appeal tool was used to assess the quality of included studies. Eligibility criteria such as study context, study participants, study setting, outcome measures, and key findings were used to identify relevant literature that measured the association between socioeconomic status and health behaviors. Out of 2,391 studies, only 46 met the final eligibility criteria and were assessed in this study. Our review found that children and adolescents with low socioeconomic status face an elevated risk of unhealthy behaviors (e.g., early initiation of smoking, high-energy-dense food, low physical activity, and involvement in drug abuse), in contrast to their counterparts. Conversely, children and adolescents from higher socioeconomic backgrounds exhibit a higher prevalence of health-promoting behaviors, such as increased consumption of fruit and vegetables, dairy products, regular breakfast, adherence to a nutritious diet, and engagement in an active lifestyle. The findings of this study underscore the necessity of implementing specific intervention measures aimed at providing assistance to families from disadvantaged socioeconomic backgrounds to mitigate the substantial disparities in health behavior outcomes in children and adolescents.

## 1. Introduction

Health behavior constitutes a fundamental facet of individuals’ holistic well-being, exerting a substantial influence on their physical health, mental well-being, and overall longevity (1–5). Over the past decades, considerable attention has been directed toward comprehending health behaviors and cultivating healthy lifestyles (6–9). However, socioeconomic status (SES) (e.g., income, education, occupation, social position) pose a significant obstacle to achieving these objectives (10). Thus, people from low-SES backgrounds are more likely to exhibit a higher rate of smoking tobacco, drinking alcohol, excess weight gain, and a sedentary lifestyle, which has been consistently identified as a pivotal determinant of premature and preventable morbidity and mortality compared to their counterparts (11–13).

The literature reveals that children and adolescents’ health and health behaviors primarily depend on their parental SES backgrounds (14–18). It is well documented that children and adolescents from high parental SES backgrounds have more access to education, housing, food, clothing, health services, and social services (19–23). These advantageous services enhance self-confidence, self-esteem, and self-efficacy, which promotes a healthy lifestyle and reduces the risk of stress in youngsters (14, 15, 17). In contrast, parental support, healthy parental lifestyle, medical services, and social networks are less open to children and adolescents with low parental SES (21, 24–27). Hence, poor availability of goods and services increases the likelihood of physical and mental health issues and encourages them to adopt risky behaviors (e.g., smoking, drinking alcohol, illicit drug use, gambling) to deal with their concerns (28–33). Therefore, parental social, cultural, and economic status are fundamental determinants of health and health behaviors in children and adolescents, which are apparent from the early stages of life and persist into adolescence (34, 35).

Globally, millions of children, particularly those with low socioeconomic profiles, do not start their lives in a healthy state (36, 37). This could be due to insufficient goods and services, which are the primary causes of impairment in children’s neuro-biological development, resulting in poor social, emotional, psychological, and physiological outcomes (38, 39). Thus, focusing on improving support for deprived and underserved populations is a powerful strategy to establish the roots of healthy behaviors in childhood and adolescent development (40). Therefore, it is recommended that every government adopt a health and health equity policy program to promote positive health behaviors among its population (41–44).

Numerous studies have been documented in the international literature to determine (45), analyze (46), and explored (47, 48) the relationship between SES and health behaviors in children and adolescents. For example, in a study conducted by Liu et al. (49), adolescents hailing from families with lower parental SES exhibited a significantly higher likelihood of [OR = 2.12, 95% CI, 1.49–3.01] cigarette smoking than those from middle and high SES backgrounds. Melotti et al. (50) explored the association between parental SES and alcohol consumption among adolescents. They discovered an inverse association, revealing that adolescents with low SES had higher odds (OR: 1.26, 95% CI, 1.05–1.52) of consuming alcohol compared to those with high SES backgrounds. Furthermore, a seminal study by Krist et al. (51) investigated the impact of parental SES on physical activity levels in children and adolescents. The results indicated that those from lower parental SES backgrounds were less likely to engage in regular physical activity (OR = 0.90, 95% CI 0.63–1.29) in comparison to their peers from higher SES backgrounds. Collectively, this extensive body of evidence underscores the pivotal role played by parental SES in shaping the multifaceted landscape of health behaviors among children and adolescents. These disparities in health behaviors often contribute to adverse health outcomes, including a heightened prevalence of chronic diseases such as obesity, diabetes, and cardiovascular diseases (52).

Given the evidence above, examining the association between parental SES and health behaviors of children and adolescents holds profound scientific significance and societal consequences. Thus, a good understanding of how parental SES influences health behaviors among young individuals enables the identification of vulnerable populations at an early stage, allowing for targeted interventions and preventive measures to combat socioeconomic comorbidities and their consequences during childhood and adolescence. However, to the best of our knowledge, the existing body of literature examining the association between SES and health behaviors (e.g., protective health behaviors and damaging health behaviors) among children and adolescents remains limited (45–47, 53, 54). To address this gap in the literature, this review comprehensively examines the association between SES and health behaviors in children and adolescents aged 3–18 years, utilizing the literature on SES and health behaviors. In this review, health behaviors were examined in two ways: (i) protecting health behaviors and (ii) impairing health behaviors among children and adolescents. Protecting health behaviors is defined as the consumption of fruit vegetables, consumption of dairy products, regular breakfast, and involvement in physical activity during leisure time (55). Consumption of high-fatty foods (e.g., chips, noodles), high sugary drinks (e.g., fruit juice, Coco Cola, cordial) and engagement in smoking (i.e., tobacco, cannabis), illicit drugs, alcohol consumption, and sedentary activities during their leisure time are defined as unhealthy behaviors exhibited by children and adularescent (56). In this review study, we were particularly interested to examining two important research questions: (i) Does low socioeconomic status influence risky or impair health behavior patterns among children and adolescents compared to their counterparts? (ii) What is the association between SES and health behaviors in children and adolescents? Through an exploration of these research questions, this study will help to better understand the relationship between SES and health behaviors in children and adolescents. By doing so, this study seeks to provide valuable insights that can inform potential policy interventions aims at enhancing the health behavior outcomes of children and adolescents, particularly those who come from underserved and disadvantaged socioeconomic backgrounds.

## 2. Methods

This review article followed the Preferred Reporting for Systematic Review and Meta-Analysis Protocol (PRISMA-P) guidelines in order to identify studies that were screened, included and excluded in this review (57). PRISMA-P helps to provide a guideline for development of protocol for systematic review, and meta-analysis in order to improve the quality and transparency of the studies (58, 59). In this review, we used the PRIMA checklist shown in Supplementary Table S3.

### 2.1. Data sources and literature search

To identify relevant articles on SES and health behaviors in childhood and adolescence, different electronic databases of various disciplines were searched, including Academic Search, APA PsycArticles, APA PsycInfo, Cumulative Index of Nursing and Allied Health Literature (CINAHL), Health Source, Nursing/Academic Edition, Psychology and Behavioral Sciences Collection, Sociology Source, Sociology Source Ultimate, PubMed, Web of Science, and Science Direct. These electronic databases were searched using the keywords: socioeconomic, “socio-economic,” “health behavior,” “health behavior,” “health behaviors,” “health behaviors,” teen*, adolescent*, and child*” (see Supplementary Table S1). The articles were subsequently exported using EndNote citation manager X9 version.

### 2.2. Criteria of included studies

In this systematic literature review, we included studies that reported an association between SES and health behaviors. Additionally, we considered studies that had at least one specific health behavior, either protecting or impairing health behaviors, such as smoking, drinking alcohol, illicit drugs, physical exercise, consumption of fruit and vegetables, and dietary habits. Consequently, all peer-reviewed prospective, retrospective, quantitative studies from both developed and developing countries, published in the English language were included. Subsequently, national representative surveys (cross-national and longitudinal) consisting of more than 500 samples from the sampled population (i.e., children and adolescents aged 3–18 years) were also included. In this study, we considered children (i.e., 3–12 years) and adolescents (13–18 years of age) as defined by the US Department of Health and Johns Hopkins Medicine, respectively (60, 61). In summary, the inclusion criteria for this review study were structured in accordance with the PICOS model, where: “P” = Population (i.e., children and adolescents aged 3 to 18 years old), “I” = intervention (we do not have intervention in this review study), “C” = comparator (i.e., high SES and low SES backgrounds), “O” = Outcome of this study (i.e., health behavior either protecting or impairing health behaviors), and “S” = study design (i.e., cross-sectional, longitudinal only).

### 2.3. Criteria of excluded studies

Based on predefined eligibility criteria, we evaluated the titles and abstracts of the identified studies. In cases where the studies were found relevant to our review, we thoroughly assessed the full text. However, if the studies were not relevant to our study, such as national income inequalities and health behavior or national per-capita income inequalities and health behaviors in childhood and adolescence, we excluded such studies. Additionally, if the study did not meet the eligibility criteria such as population, comparator, outcome, and study design, we excluded the paper from this study. For example, review articles, pilot studies, reports, dissertations, books, symposia, supplementary, prospective, or intervention studies, and articles published in other languages. Articles published before 2000 were also excluded from this study, primarily because their full-text versions were not readily accessible. Furthermore, articles with a sample size of less than 500 were excluded from the analysis. The decision to exclude studies with a sample size less than 500 was driven by a combination of methodological constraints. Two articles had sample sizes of 246 and 310, respectively; however, these studies found no association between SES and health behaviors in adolescents. Therefore, we decided to exclude them from the final analysis.

### 2.4. Data extraction

The data extraction was done by two independent reviewers using a data extraction table. The data extraction was based on the Joanna Briggs Institute (JBI) checklist (e.g., country, study setting/context, participant characteristics, outcome measures, and key findings). The final data extraction was based on the phase two screening of studies (i.e., 46 studies) by NG following the PRISMA guidelines (57), while GD, MMR, and RK approved data extraction.

### 2.5. Quality assessment of study

The Joanna Briggs Institute (JBI) and System for the Unified Management of the Assessment and Review of Information (SUMARI) is an appraisal tools assist in evaluation of the trustworthiness outcomes of the included studies (62–64). For the cross-sectional studies, JBI SUMARI apprise as follows: (i) inclusion criteria were clearly defined for the study population; (ii) study subject and study setting were clearly explained; (iii) exposures were scientifically measured; (iv) standard criteria and objectives were used to assess the measurement of the study; (v) confounding factors were identified; (vi) a strategic plan was developed to address the confounding factors; (vii) the results of the studies were reliable and valid; (viii) an appropriate statistical method was used to analyze the study. On the other hand, cohort studies were evaluated as follows: (i) the sample was recruited from the study population; (ii) exposures were measured equally between the exposed and unexposed groups; (iii) exposures were measured in a valid and reliable way; (iv) confounding factors were identified; (v) the statistical plan was used to address the confounding factors; (vi) at the beginning of the study, the sampled population was free from exposures; (vii) outcomes of the study were measured in a valid and reliable way; (viii) follow-up time was sufficiently reported; (ix) follow-up was completed, and if not, reasons were well explained; (x) plans were explored to address the issue of those in the sample lost to follow-up; (xi) the standard statistical method to analyze the study was used. To establish the credibility of our findings, every included study was apprised using the JBI SUMARI and provided the score for each study by two independent reviewers. Studies were included in this review if they had gained a score of 60 percent or above, while studies with a score of less than 60 percent were excluded. However, in the context of this study appraisal, none of the papers met the criteria for a score below 60 percent. As a result, it was not necessary to exclude any studies through the application of the JBI SUMARI appraisal tools (see Supplementary Table S4). In alignment with these principles, our review study also employed the JBI SUMARI to contribute to the overall reliability and quality of the review findings (65, 66).

Furthermore, the risk of bias in the included studies was evaluated using the 10-item grading scale for prevalence studies developed by Hoy et al. (67). The study methodology, case definition, prevalence periods, sampling, data collection, reliability, and validity of the investigations were thoroughly scrutinized. Each study was categorized as exhibiting either a low risk of bias (shown by affirmative replies to domain questions) or a high risk of bias (indicated by negative responses to domain questions). In each study, a binary scoring system was used to assign a value of 1 (indicating presence) or 0 (indicating absence) to each domain. The cumulative sum of these domain values was used to derive the overall study quality score. The assessment of bias risk was performed by calculating the total number of high-risk biases in each study. Studies were categorized as having a low risk of bias if they had two or fewer high-risk biases, moderate risk of bias if they had three or four high-risk biases, and high risk of bias if they had five or more high-risk biases. To resolve discrepancies among the reviewers, a consensus-based approach was employed for the final categorization of the risk of bias (see Supplementary Table S5).

### 2.6. Outcome measurement of the study

The outcomes of this study were health behaviors, either through health-protective behaviors (e.g., consumption of fruit and vegetables, consumption of a healthy diet, physical exercise) or impairing health behaviors (e.g., smoking, drinking alcohol, high sedentary lifestyle and illicit drug use), in children and adolescents (68). Childhood and adolescence are two distinct stages of life that are characterized by diverse features in the respective age groups (e.g., physical and psychological) (69, 70). Childhood is a time of rapid and remarkable development, encompassing significant strides in the physical, cognitive, social, and emotional dimensions that outpace the progress seen in other life stages. During this period, children’s innate intellectual curiosity flourishes, driving them to explore the world around them and actively engage in interactive experiences (71, 72). On the other hand, adolescence is widely acknowledged as a transformative phase known as the “storm and stress” period, characterized by profound changes in biological, cognitive, psychosocial, and emotional realms (73). These transformative shifts often give rise to a range of adjustment challenges, including mood swings, propensity for risk-taking behaviors, and conflicts with both parental figures and peers (73). Thus, considering the relationship between health behaviors in childhood and adolescence is pivotal, therefore, this review study examined the association between parental SES and health behaviors in children and adolescents separately.

### 2.7. Measure of socioeconomic status (SES)

SES is a multidimensional construct and is measured objectively by income, education, and occupation, or subjectively by prestige, place of residence, ethnic origin, or religious background, covering both objective and subjective measures of SES (74–76). The stratification of SES into subgroups (e.g., low SES and high SES) was predicted based on goods and materials consumed by the individual in a household, including durable goods such as televisions, bicycles, and vehicles, as well as housing-related attributes such as access to drinking water, food, bathroom facilities, and agricultural and flooring materials (77). Households that possessed adequate provision of food, water, hand-washing materials, agricultural products, fields for production, and other consumer items for a duration of merely 6 months were categorized as having low-income or low- SES backgrounds. On the other hand, families that possessed an ample supply of food and other products, including consumer goods, to sustain themselves for a period of 12 months or more were classified as affluent or wealthy or high SES backgrounds (77, 78). Based on previous literature (21, 22, 77, 79, 80), our study defined low SES and high SES backgrounds and examined the association between SES and health behaviors in children and adolescents.

## 3. Results

### 3.1. Description of the study

**Figure 1:**
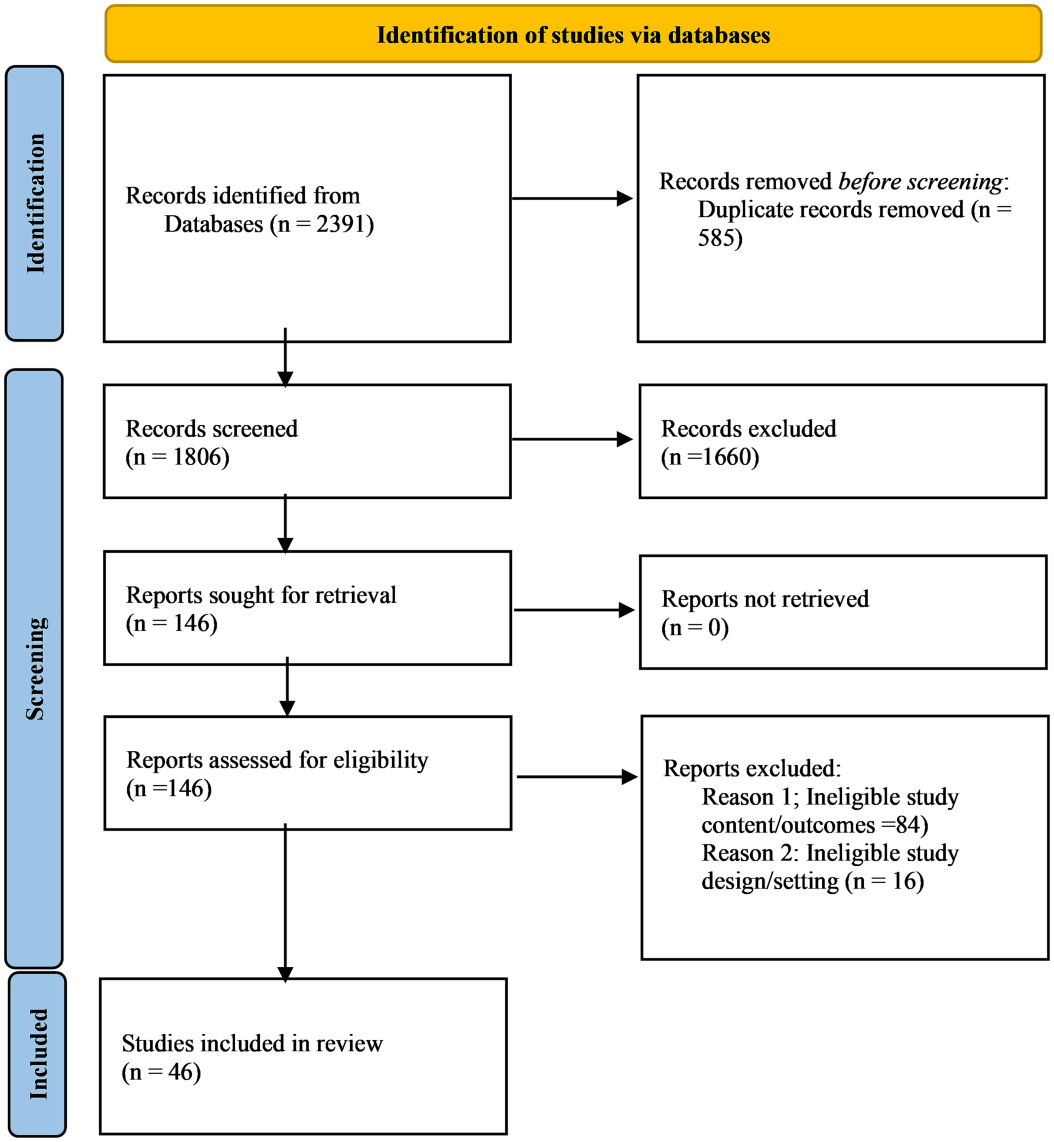
PRISMA flow diagram.

In this systematic literature review, 2,391 articles were initially retrieved, and 585 duplicates were removed before entering the first phase of screening. A total of 1806 articles were assessed in the first phase of screening based on their titles and abstracts. Thus, in the first screening phase, we retained only 146 articles and rejected 1,660 articles. Consequently, in phase two of the screening, we assessed 146 papers based on their full text. Of these, only 46 articles met the inclusion criteria for further evaluation. Hence, these 46 articles were eligible in our final review, which is shown in Figure 1.

A total of 46 articles were assessed to examine the association between SES and health behaviors in childhood and adolescence. Most of the studies were from Europe (*n* = 36), followed by Asia (*n* = 9), North America (*n* = 5), and Africa (*n* = 2). There has been a noticeable increase in studies on SES and health behavior in childhood and adolescence in the past decade (i.e., 2010 to 2020). From a total of 46 studies, 13 studies (79, 81–92) were published between 2000 to 2010, while 33 studies (25, 47–51, 53, 93–118) were published from 2011 to 2022.

### 3.2. Parental socioeconomic status and smoking

#### 3.2.1. Parental socioeconomic status and smoking among children

From a total of 46 studies, 18 studies (25, 47, 49, 50, 53, 79, 81, 84, 86–88, 93, 96, 97, 104, 105, 107, 109) showed an association between SES and smoking behavior in children and adolescents. Out of 18 studies, five reported a positive association between low SES and smoking behavior in children, implying that children with low SES had a greater risk of exposure to early smoking, and experimenting with smoking, compared to those who were from a high SES background (25, 49, 53, 104, 105).

#### 3.2.2. Parental socioeconomic status and smoking among adolescents

From a total of 18 studies, 12 studies showed a positive association between low SES and smoking behaviors in adolescents, indicating that adolescents with low SES had a higher risk of smoking behaviors than those with a high SES background (50, 79, 81, 84, 86–88, 93, 96, 97, 107, 109), while one study found a negative association between low SES and smoking behavior (47). In summary, a majority of 17 studies (94.44%) supported that children (5 studies, 27.77%) and adolescents (12 studies, 66.66%) from low SES backgrounds were more likely to be exposed to, have tried, or smoked daily, compared to those with high SES (see Table 1).

**Table 1:** Studies examining the association between parental SES and smoking.

SN	Authors	Methodology description						Findings	Key strengths and limitations	Quality score
		Study design	Sample size (n)	Continent/country	Age range	SES measure	Method of analysis			
1	Moore and Littlecott (2015)	Cross-sectional, National representative	9,194	Wales	11–16 years	SES	The mixed-effects logistic regression model	Adolescents from high SES were less likely (OR: 0.88, 95% CI, 0.81 to 0.95) to smoke than those from low SES.	High parental SES was linked to healthier behaviors in children and adolescents. Yet, this cross-sectional study, potential self-reporting bias, and the lack of established causation limit definitive conclusions regarding the high SES and reduced adolescent smoking relationship.	8
2	Liu et al. (2016)	Cross-national study	3,690	China and Finland	11–15 years	SES	Logistic regression	In China, low SES adolescents (boys: OR 2.12, 95% CI 1.49–3.01; girls: OR 1.07, 95% CI 0.64–1.81) were more likely to ever smoke compared to those from middle/high SES. In Finland, low SES adolescents (boys: OR 1.17, 95% CI 0.77–1.79; girls: OR 1.68, 95% CI 1.07–2.65) were more likely to smoke weekly than their middle/high SES counterparts.	Social, economic, and cultural factors influence smoking behaviors among children and adolescents. However, this study used only FAS as the SES measure due to limited common indicators between Chinese and Finnish surveys, suggesting a need to include perceived family wealth for cross-country comparisons.	8
3	Simetin et al. (2013)	Cross-sectional, national representative	1,601	Croatia	15 years	SES	Multi-level logistic regression	Adolescents from high SES were 1.56 times more likely to smoke than those from medium and low SES.	Disposable income, different peer influences, parental attitudes, and access to resources are associated with smoking in adolescents. However, the association between socioeconomic factors and risk behaviors may be influenced by adolescents’ relative resilience to socioeconomic inequalities.	8
4	Levin et al. (2014)	Cross-sectional, National representative	2,692	Scotland	15 years	SES	The multilevel logistic regression model	Low SES adolescents had higher odds of smoking, current smoking, daily smoking (Boys: OR smoking 1.58, 95% CI 0.97–2.56; OR current smoking 1.69, 95% CI 0.87–3.29; OR daily smoking 1.82, 95% CI 0.82–4.03; Girls: OR smoking 1.26, 95% CI 0.97–1.64; OR current smoking 1.63, 95% CI 1.18–2.25; OR daily smoking 1.56, 95% CI 1.03–2.25) compared to high SES peers. Low SES adolescents (boys and girls) were also more likely to smoke weekly (OR boys: 1.14, 95% CI 1.00–1.31; OR girls: 1.17, 95% CI 1.02–1.32) than those from high SES.	Lack of educational awareness, and cultural factors contribute to smoking behaviors. However, a study has a low sample size for geographic comparison in ever smoking. Therefore, results might not be conclusive.	8
5	Richter et al. (2009a)	Cross-national survey	97,721	33 countries consisting of Asia (*n* = 1), Europe (*n* = 30), and North America (*n* = 2)	13–15 years	SES	The multilevel logistic regression model	Adolescents from low SES backgrounds had higher weekly smoking rates (Boys: OR 1.14, 95% CI 1.00–1.31; Girls: OR 1.17, 95% CI 1.02–1.32) compared to their high SES counterparts.	Economic stress and parental smoking behaviors are more common in low SES backgrounds. However, interpreting adolescent behavioral patterns obtained from self-reports can be challenging due to the potential influence of social desirability bias, especially in the case of health behaviors like smoking.	8
6	Doku et al. (2010)	Longitudinal	96,747	Europe (Finland)	12–18 years	Parental occupation, parental educational level	Logistic regression	Adolescents (boys and girls) with fathers in blue-collar occupations had a higher likelihood of smoking (Boys: OR 1.3, 95% CI 1.2–1.4; Girls: OR 1.4, 95% CI 1.3–1.5). Similarly, adolescents (boys and girls) with lower levels of maternal education had a higher chance of smoking (Boys: OR 1.5, 95% CI 1.3–1.6; Girls: OR 1.5, 95% CI 1.4–1.7) compared to those with high maternal educational levels.	Lower occupations and educational levels are often associated with smoking behaviors in children and adolescents. However, this study has a higher non-response rate.	8
7	Richter et al. (2009)	Cross-national	86,667	Europe (*n* = 26) North America (*n* = 2)	13–15 years	Parental occupation, and SES	Logistic regression	European adolescents (from West, North, South, Central, and East regions) with low SES and parental occupation were more likely to smoke compared to those with high or middle SES. Similarly, adolescents in Canada and the USA with low SES had a higher likelihood of smoking than their high or middle-SES peers, particularly when their parents had lower occupational status	Adolescent smoking behaviors were largely determined by low parental SES backgrounds. However, methodological constraints limit findings.	9
8	Poulain, et al. (2019)	Longitudinal	992	German	3–18 years	SES	Mixed-effect model	Children and adolescents aged 10 to 18 with mothers having higher education (OR: 0.86, 95% CI 0.74–0.99), higher occupational status (OR: 0.61, 95% CI 0.47–0.79), and high SES (OR: 0.77, 95% CI 0.58–1.02) had a reduced likelihood of smoking compared to their peers.	Educational influence and role models provide their children with accurate information about the health consequences of smoking. However, this study potentially lacks representativeness in the distribution of socioeconomic classes which may restrict the applicability of the study’s results to the broader population.	8
9	Melotti et al. (2011)	Longitudinal	5,699	UK	13 years	Maternal education and parental social status	Multiple logistic regression model	Adolescents with mothers lacking education, lower social class, or low family income had higher odds of ever smoking (OR: 1.14, 95% CI 0.95–1.38; OR: 1.15, 95% CI 0.81–1.60; OR: 1.17, 95% CI 0.92–1.48) compared to their counterparts.	Limited awareness and education associated with risks associated with smoking in adolescents. However, the study has large missing data which might influence the findings.	9
10	Moor et al. (2015)	Cross-national	52,709	35 countries (33 in Europe, North- America and Israel)	15 years	SES	Multilevel regression model	Adolescents with low parental SES had a higher risk of smoking compared to those with higher SES, with more pronounced differences among girls (Boys: OR 1.14, 95% CI 1.05–1.23; Girls: OR 1.36, 95% CI 1.26–1.46).	Social class and peer influence increases the likelihood of smoking. The study measures family SES by material consumption. This may lead to the misclassification of parental social class.	10
11	Park and Hwang (2017)	Cross-sectional	72,435	South-Korea	13–18 years	SES	Multivariate logistic regression	Adolescents from low parental SES had a slightly higher chance of smoking (OR: 1.027, 95% CI 0.929–1.136) compared to those from high parental SES.	Economic instability and stressful events contribute to an increased probability of engaging in smoking behavior. However, the study has some methodological constraints.	8
12	Johansen et al. (2006)	Cross-sectional	3,458	Denmark	14–16 years	Maternal occupation	GEE logistic regression	Adolescents with unemployed mothers had a slightly higher risk of daily smoking (OR: 1.10, 95% CI 0.77–1.56) compared to their counterparts.	Parental unemployment can increase the probability of smoking in a family, including their children. However, the study did not provide a clear pattern of how SES contributes to smoking.	8
13	Kislitsyna et al. (2010)	Cross-sectional	815	Russia	12–17 years	SES	Logistic regression model	Adolescents with lower parental SES had higher odds of smoking, particularly among girls (Boys: OR: 1.31, 95% CI 0.73–2.35; Girls: OR: 4.08, 95% CI 1.13–14.69), compared to those from higher parental SES.	Higher parental education helps to reduce smoking behaviors in children and adolescents. However, this study underreports the smoking data and leads to inconclusive of findings.	6
14	Doku et al. (2010)	Cross-sectional	1,165	Ghana	13–18 years	Parental income and parental education	Logistic regression	Adolescents with low parental income had a higher smoking risk (OR: 2.2, 95% CI 0.9–5.3) than those from high parental SES. Likewise, those with lower parental education (illiterate) faced a higher smoking risk (OR: 3.0, 95% CI 1.3–7.3) compared to those with high parental education (tertiary).	Income plays a crucial role in adjusting the behaviors of individuals including children and adolescents. However, cause and effect cannot be emphasized as etiological conclusions.	7
15	Simetin et al. (2011)	Cross-sectional.	3,296	Croatia	11–15 years	SES	Binary logistic regression	Children and adolescents with high parental SES had a reduced smoking likelihood (Children: OR 0.4, 95% CI 0.1–1.2; Adolescents: OR 0.8, 95% CI 0.6–1.1) compared to those with low parental SES.	High social class and prestige help to adopt healthy behaviors and reduce the chances of smoking. Self-reporting bias and lack of causation limit conclusions on the high SES and smoking relationship.	6
16	Lazzeri et al. (2014)	Cross-sectional	3,291	Italy	11–15 years	Parental income	Logistic regression	Children and adolescents with high parental income had a reduced smoking likelihood (Children: OR 0.28, 95% CI 0.030–2.40; Adolescents: OR 0.92, 95% CI 0.50–1.67) compared to those with low parental income.	High SES contributes to reducing smoking behaviors in children and adolescents. However, this study also used the self-reported questionnaire, which may introduce inaccuracies that affect statistical connections.	6
17	Sweeting and Hunt (2015)	Cross-sectional	2,503	Scotland	13–15 years	SES	Logistic regression	Adolescents with low parental SES had higher odds of ever smoking and weekly smoking (OR ever smoke: 1.27, 95% CI 0.91–1.78; OR weekly smoke: 1.37, 95% CI 0.91–2.05) compared to those with high parental SES.	The pattern of smoking behaviors is based on parental SES background. However, methodological constraints affect the findings of this study.	6
18	Pförtner et al. (2015)	Cross-national survey	6,511	Belgium, Canada, England, Romania	15 years	SES	Pooled logistic regression models	Adolescents from low SES had a higher chance of smoking (OR: 1.44, OR 95%: CI 1.19–1.83) than those from medium and high SES.	The difference in smoking prevalence is determined by SES. Despite the statistical significance, this study has effect sizes that raise doubts about the significance of these findings.	8

### 3.3. Parental socioeconomic status and drinking alcohol

#### 3.3.1. Parental socioeconomic status and drinking alcohol among children

Out of 46 studies, 18 studies (25, 47, 50, 53, 79, 83–85, 91, 97, 100–102, 104, 105, 107, 108, 115) assessed the association between SES and drinking alcohol. Of them, seven studies reported a positive association between high SES and drinking alcohol in children (25, 53, 83, 102, 104, 105, 115). This implies that younger children from higher SES backgrounds are more likely to experiment with drinking alcohol compared with their counterparts. One study found a negative association between drinking alcohol and high SES (100).

#### 3.3.2. Parental socioeconomic status and drinking alcohol among adolescents

With regard to SES and drinking alcohol by adolescents, from 18 studies, six reported a negative association between drinking alcohol and high SES (50, 85, 91, 101, 107, 108). Conversely, three studies reported a positive association between drinking alcohol and high SES (47, 79, 97). This positive association indicates that adolescents from a high SES background were found to have a higher chance of drinking alcohol than their counterparts. Moreover, a study by Richter et al. (84) reported that adolescents from Western, Northern, Southern (except Spain), Central and Eastern (except Latvia) Europe, and from a low SES background, were found to have a lower chance of drinking alcohol compared to their counterparts; however, adolescents from Scotland, Wales, Norway, Finland, Denmark, Greece, Italy, Malta, Hungary, Russia, and Ukraine were only statistically significant. In summary, out of 18 studies, children (seven studies, or 38.88%) and adolescents (three studies, or 16.66%) from a high SES background had a higher chance of drinking alcohol than those from a low SES background, while six studies (33.33%) found a negative association between a high SES background and drinking alcohol in adolescents (Table 2).

**Table 2:** Studies examining the association between parental SES and alcohol consumption.

SN	Authors	Methodology description						Findings	Key strengths and limitations	Quality score
		Study design	Sample size (n)	Continent/country	Age range	Measure	Method of analysis			
1	Moore and Littlecot (2015)	Cross-sectional	9,194	Wales	11–16 years	SES	The mixed-effects logistic regression model	Children and adolescents with high SES were found to be at a greater risk of drinking alcohol than those who were from low SES.	Families with higher SES backgrounds provide a suitable home environment for drinking alcohol. However, this cross-sectional study lacks to established causal association between the high SES and lower teenage smoking association.	8
2	Simetin et al. (2013)	Cross-sectional	1,601	Croatia	15 years	SES	Multi-level logistic regression	Adolescents from high SES had a higher chance of drunkenness (OR: 1.446; S.E.: 0.16) compared to those from low SES.	Predominantly, higher parental social status increases the probability of drinking alcohol in children and adolescents. However, adolescents’ resilience to socioeconomic inequalities may affect the relationship between socioeconomic characteristics and risk behaviors.	8
3	Richter et al. (2009)	Cross-national	86,667	Europe (*n* = 26) North America (*n* = 2)	13–15 years	SES, parental occupation	Logistic regression	Low SES adolescents in most European regions had lower alcohol consumption rates, except in Spain and Latvia. Conversely, low SES North American adolescents, except in Canada, had higher alcohol consumption rates. Adolescents in Southern, Northern, and Western Europe with low parental occupation levels showed less alcohol consumption, except in Ireland and Wales. In contrast, adolescents from Central Europe, Eastern Europe, and North America (excluding Canada) with low parental occupation levels had a higher likelihood of alcohol consumption compared to high-occupation level counterparts.	Interestingly, low parental SES and low level of occupation level enhance the heavy alcohol drinking problem in adolescents. However, methodological constraints limit conclusive results.	8
4	Liu et al. (2013)	Cross-national	3,690	China and Finland	11–5 years	SES	Logistic regression	High SES children and adolescents were more likely to initiate alcohol use at an early age (Girls: OR 1.55; Boys: OR 1.92) compared to their counterparts.	HIGH-SES adolescents are more likely to consume alcohol under parental supervision. However, self-reporting questionnaires related to alcohol use may be biased due to social desirability.	8
5	Poulain et al. (2019)	Longitudinal	992	German	3–8 years	SES, maternal education, maternal occupation	Mixed-effect model	Children and adolescents with highly educated mothers (OR: 1.04, 95% CI 0.91–1.18) and mothers in high-occupational positions (OR: 1.01, 95% CI 0.83–1.23) were more likely to engage in alcohol consumption compared to those with lower maternal education and occupation levels.	The availability of alcohol at home for various reasons (i.e., cultural activities, and celebrations) increased the chance of drinking alcohol. However, this study may have limited representativeness across socioeconomic classes, potentially limiting its broader applicability.	9
6	Melotti et al. (2011)	Longitudinal	5,699	UK	13 years	Family income, maternal education, occupation	Multiple logistic regression models	Adolescents with illiterate mothers had higher odds of alcohol consumption (OR: 1.26, 95% CI 1.05–1.52) compared to those with literate mothers. Similarly, adolescents from lower social class backgrounds also had higher odds of alcohol consumption (OR: 1.22, 95% CI 0.88–1.71). Conversely, lower family income was associated with lower odds of alcohol consumption (OR: 0.87, 95% CI 0.68–1.11)	Lower literacy levels and awareness seem to be effective in increasing the consumption of alcohol in adolescents. However, the study exhibits significant gaps in data, potentially impacting the validity and reliability of the conclusions.	10
7	Richter et al. (2006)	Cross-national	142,868	28 countries, including United State of America	11–15 years	SES	The multivariate logistic regression model	Low parental SES was associated with a reduced risk of drunkenness in children and adolescents across most European countries. However, boys from low parental occupation levels had a higher risk of drunkenness in several countries, while girls had a higher risk in most countries with low parental occupation, compared to their counterparts in high parental occupation.	There is a difference in accessing the resources. Indicating that alcohol is expensive, and lower-income families may prioritize basic needs over alcohol purchases. So, adolescents from low SES backgrounds are less likely to consume alcohol than those from high SES backgrounds. However, several methodological aspects limit the explanatory capacity of these results	8
8	Park and Hwang (2017)	Cross-sectional	72,435	South-Korea	13–18 years	SES	Multivariate logistic regression	Adolescents from low SES backgrounds had significantly lower alcohol consumption (OR: 0.809, 95% CI 0.782–0.869) compared to their counterparts.	The level of health literacy seems to decline in the consumption of alcohol in individuals. Thus, adolescents from low SES backgrounds had lower levels of health literacy and were more prone to consume alcohol compared with their counterparts. Nevertheless, there are certain methodological limitations in the study	8
9	Andersen et al. (2007)	Cross-sectional	1,302	Denmark	15 years	Parental social class	Multivariate logistic regression	Adolescents from high parental social class backgrounds were less likely to consume alcohol (Boys: OR: 0.53, 95% CI 0.28–1.01; Girls: OR: 0.55, 95% CI 0.27–1.13) compared to those from low parental social class.	Social prestige seems to be effective in decreasing the consumption of alcohol in adolescents. However, it did not provide a causal relationship between SES and drinking alcohol behavior.	8
10	Johansen et al. (2006)	Cross-sectional	3,458	Denmark	14–16 years	Maternal occupation	GEE logistic regression	Adolescents with unemployed mothers had a significantly reduced risk of weekly alcohol consumption (OR: 0.48, 95% CI 0.34–0.68) compared to their counterparts.	Being unemployed often means having limited financial resources. So, those who were from low SES backgrounds seemed to consume less alcohol compared with their counterparts. However, the study failed to establish a causal association between SES and drinking alcohol behavior.	6
11	Melotti et al. (2013)	Longitudinal	6,170	United Kingdom	11 years	Maternal education	Multivariable logistic regression	Children with mothers who had higher education were less likely to start drinking alcohol early (OR: 0.91, 95% CI 0.84–0.99) compared to their counterparts.	Educated individuals may have a better understanding of the potential negative consequences of alcohol consumption at a young age. Thus, those adolescents belonging to high parental education levels were found to have low alcohol consumption in their children. However, there might be a chance of reporting bias. So findings may be inconsistent.	10
12	Doku et al. (2012)	Cross-sectional	1,195	Ghana	12–18 years	SES	Logistic regression	Adolescents with low parental SES were found to have more drunkenness (OR: 2.3, 95% CI, 1.4–3.9) compared to those with high parental SES.	Adolescents have more free time and fewer constructive ways to spend it, potentially leading to alcohol use. However, data was collected using self-report measures and utilized a cross-sectional research design. So, it cannot establish a causal association and there might be a chance of biases in the findings.	7
13	Simetin et al. (2011)	Cross-sectional.	3,296	Croatia	11–15 years	SES	Binary logistic regression	Children and adolescents with high parental SES were found to have a higher chance of drunkenness (OR: 1.1, 95% CI 0.7–1.7) compared to those with low parental SES.	Social opportunities and the availability of alcohol at home seem to increase the chance of drinking alcohol in children and adolescents. Self-reporting bias and lack of causation limit conclusions on the high SES and drinking alcohol.	8
14	Pedroni et al. (2021)	Cross-sectional	4,364	Belgium	10–14 years	SES	Pearson’s chi-square tests and logistic regression	Children and adolescents from low parental SES were less likely to have consumed alcohol (Boys: OR 0.56, 95% CI 0.32–0.98; Girls: OR 0.71, 95% CI 0.40–1.23) compared to their high SES counterpart	Not all adolescents from low SES backgrounds will abstain from alcohol, and many factors, including personal choices, peer influences, and cultural contexts, can influence outcomes. However, the findings did not provide sufficient evidence to establish a causal relationship.	7
15	Lazzeri et al. (2014)	Cross-sectional	3,291	Italy	11–115 years	SES	Logistic regression	Children from households with high parental income had a higher likelihood of alcohol consumption (Children: OR 1.27, 95% CI 0.68–2.37) compared to those from low-income households. Conversely, adolescents with high parental income had a lower likelihood of alcohol consumption (Adolescents: OR 0.71, 95% CI 0.52–0.96) compared to their low-income counterparts.	High parental income often provides children with greater financial resources, allowing them to afford alcohol or attend social events where alcohol is readily available. However, the self-reported questionnaire in this study may contain biases that influence the conclusions.	6
16	Sweeting and Hunt (2015)	Cross-sectional	2,503	Scotland	13–15	SES	Logistic regression	Adolescents with low parental SES were significantly found higher chance of ever drinking alcohol (OR: 1.18,95% CI, 0.69–2.01) compared to those with high parental SES.	Maybe some lower-income communities may contribute to a higher acceptance of alcohol consumption, as a social norm or coping mechanism. However, these results were influenced by potential bias.	9
17	Pape et al. (2018)	Cross-sectional	12,966	Norway	14–17 years	Parental education	Poisson regression	Adolescents with low parental education were found to have a higher chance of drinking alcohol (RR: 1.42, 95% CI 1.24–1.62) compared to their counterparts.	Adolescents with low parental education may have fewer opportunities for extracurricular activities. This can lead to more idle time and an increased likelihood of experimenting with alcohol. However, the findings were influenced by the way of measuring the drinking alcohol leading to unusual results.	8
18	Andersen et al. (2008)	Longitudinal	729	Denmark	15 years	SES	Multivariate logistic regression model	Adolescents from low SES were more likely to drink alcohol (Boys: OR: 1.67, 95% CI, 0.76–3.65) and (Girls: OR: 1.30, 95% CI, 0.47–3.58) than those from high SES.	Adolescents from families with low SES face additional stressors related to financial instability or other challenges. So, adolescents may turn to alcohol as a way to cope with these stressors. However, methodological constraints might influence the results of the study.	10

### 3.4. Parental socioeconomic status and physical activity (PA)

#### 3.4.1. Parental socioeconomic status and physical activity (PA) among children

We identified 15 studies (25, 48, 51, 53, 81, 89, 92, 94, 97, 98, 104, 105, 110, 113, 117) that examined the association between SES and PA. From a total of 15 studies, seven found a positive association between high SES and PA in children (25, 48, 51, 98, 104, 110, 117); however, two reported a negative association between high SES and PA (53, 105). This negative association implies that those children from a high SES background had a lower chance of being physically active compared to their counterparts.

#### 3.4.2. Parental socioeconomic status and physical activity (PA) among adolescents

Five studies found a positive association between high-SES and physical activity in adolescents (89, 92, 94, 97, 113). These findings implied that the adolescents from high parental SES backgrounds were more physically active compared to their counterparts, while one study found a negative association between high SES and PA (81). In summary, children from high-SES backgrounds in seven studies (46.66%) and adolescents in five studies (33.33%) had a higher chance of being more physically active than those from low- SES backgrounds (Table 3).

**Table 3:** Studies examining the association between parental SES and physical activity (PA).

SN	Authors	Methodology description								
		Study design	Sample size (n)	Continent/country	Age range	SES measure	Method of analysis	Findings	Key strengths and limitations	Quality score
1	Moore and Littlecott (2015)	Cross-sectional	9,194	Wales	11–16 years	SES	Mixed-effects logistic regression models	Adolescents from lower SES backgrounds exhibited a higher likelihood of engaging in physical activity (OR: 1.13, 95% CI 1.08, 1.18) compared to their peers from higher SES.	Lower SES backgrounds might have fewer opportunities for indoor entertainment or screen time due to limited access to electronic devices, television, or gaming consoles. However, inappropriate measures of the data and methodological constraints make inconclusive statements about the relationship between high SES and physical activity.	8
2	Hankonen et al. (2017)	Cross-sectional	659	Finland	16–19 years	SES	COM-B model	Adolescents with a lower SES were observed to face an elevated risk of physical inactivity in comparison to their counterparts with a higher SES.	Financial instability or housing insecurity can contribute to increased sedentary behaviors and decreased motivation for physical activity. However, the utilization of self-report measures to assess behavior, abilities, and environmental factors is susceptible to bias.	8
3	Richter et al. (2009a)	Cross-national survey	97,721	Europe (*n* = 30), Asia (*n* = 1), North America (*n* = 2)	13–15 years	SES	The multilevel logistic regression model	Adolescents with a lower SES demonstrated a significantly higher likelihood of engaging in physical activity when compared to their peers from higher SES backgrounds, with odds ratios of (OR: 1.34, 95% CI, 1.26–1.43) for boys and (OR: 1.57,95% CI, 1.45–1.71) for girls.	Lower SES backgrounds might be more likely to walk or bike to school or other destinations due to limited access to private transportation, thereby increasing their overall physical activity levels. However, the findings were influenced by the way physical activity is measured or reported leading to unusual results.	8
4	Yannakoulia et al. (2016)	Cross-sectional, National representative	11,717	Greece	3–18 years	SES	Classification–regression tree analysis (CART) model.	Children and adolescents from higher SES backgrounds tended to dedicate more time to sports activities, with children spending an average of 3.3 h (SD 1.9) compared to 2.7 h (SD 2.4) per week, and adolescents engaging for an average of 4.5 h (SD 3.2) versus 3.1 h (SD 3.0) per week for those from lower SES.	Higher SES communities may have better access to sports facilities, parks, and recreation programs, creating a supportive environment for sports participation. However, a low response rate might distort the findings	8
5	Poulain et al. (2019)	Longitudinal	2,492	German	3–18 years	SES	Mixed-effect model	Adolescents with mothers having higher SES had greater odds of being physically active (OR: 1.83, 95% CI: 1.45–2.31) than those with lower SES. Similarly, children with higher SES (OR: 1.42, 95% CI: 1.14–1.77) were more engaged in physical activities than those with lower SES.	Families may place a stronger emphasis on physical fitness and active lifestyles. However, this study may have limited representativeness across socioeconomic classes, potentially limiting its broader applicability.	9
6	Yang (2021)	Cross-sectional	1,040	South Korea	10–11 years	SES	Multiple linear regression model	Children from higher parental SES engaged in more weekly physical activity (β: 0.08, *p* < 0.05) than those from lower parental SES	Parenting role modeling may serve as positive role models for their children. However, this study limits the causal inferences.	8
7	Krist et al. (2017)	Cross-sectional	1,523	Germany	12–13 years	SES	A generalized linear mixed model with a logit	Children and adolescents from low parental SES had lower odds of physical activity (OR low: 0.90, 95% CI: 0.63–1.29) than those from high parental SES	Lack of role models may be fewer visible role models who prioritize and engage in physical activity. However, due to the structure of the questionnaire, the findings were overestimated.	8
8	Park and Hwang. (2017)	Cross-sectional	72,435	South-Korea	13 to 18 years	SES	Multivariate logistic regression	Adolescents with low parental SES had a higher risk of no physical activity (OR: 1.425, 95% CI: 1.336–1.521) than those with high parental SES	financial instability can contribute to increased sedentary behaviors and decreased motivation for physical activity. Nevertheless, there are certain methodological limitations in the study	8
9	Henriksen et al. (2016)	Cross-sectional	6,269	Denmark	11–15 years	Parental social class	Logistic regression	Children and adolescents from low parental social class face a higher risk of physical inactivity (OR: 2.10, 95% CI: 1.39–3.18) than those from high parental social class.	Time Constraints which can limit the time available for physical activities. However, this phenomenon is not conducive to casual interpretations.	6
10	de Buhr and Tannen (2020)	Cross-sectional	4,294	German	6–13 years	SES	Spearman’s Rho correlations	Children from higher parental SES were more physically active (r: 0.079, 95% CI: 0.025–0.132) than those from lower parental SES.	Education and awareness seem to a protective factor and make them more physically active. However, interpreting adolescent PA patterns obtained from self-reports can be challenging due to the potential influence of social desirability bias.	8
11	Pavon et al. (2010)	Cross-sectional	3,259	Nine European countries (Sweden, Greece, Italy, Spain, Hungary, Belgium, France, Germany, and Austria)	12–17 years	SES	One-way analysis of covariance	Adolescents with high parental SES exhibited significantly better physical fitness (p < 0.05) than those with low parental SES.	Access to resources appears to have a positive impact on promoting physical activity. However, methodological issues might to a risk of inconclusive findings.	6
12	Falese et al. (2021)	Cross-sectional	10,510	Six European cities (Namur, Tampere, Hannover, Latina, Amersfoort, and Coimbra).	14–17 years	Parental income and parental education	The multilevel multivariable linear regression model	Adolescents with higher parental education levels were more likely to engage in more vigorous physical activity (OR: 2.7, 95% CI: 0.3–5.1), and those with higher parental income had increased physical activity (OR: 4.7, 95% CI: 2.8–6.6) compared to their counterparts.	Awareness and knowledge lead them to encourage themselves to be physically active. However, Self-reported measures of physical activity intensity may lead to either overestimation or underestimation	8
13	Simetin et al. (2011)	Cross-sectional	3,296	Croatia	11–15 years	SES	Binary logistic regression	Children and adolescents with high parental SES had greater odds of engaging in physical activity (OR children: 1.8, 95% CI: 1.3–2.5; OR adolescents: 1.3, 95% CI: 0.9–1.8) than those with low parental SES	Access to resources and facilities increases the likelihood of physical activity. However, self-reporting bias and lack of causation limit conclusions on the high SES and PA.	6
14	Al Sabbah et al. (2007)	Cross-sectional	8,885	Palestine	12–18 years	Maternal education	Logistic regression	Adolescents with high maternal education were more likely to engage in more physical activities (OR: 1.26, 95% CI: 1.09–1.46) than those with low maternal education.	Health literacy encourages them to promote physical activity for a healthy lifestyle. Nevertheless, self-reported measurements of physical activity might result in either an overestimation or underestimation.	6
15	Lazzeri et al. (2014)	Cross-sectional	3,291	Italy	11–15 years	Parental income	Logistic regression	Children and adolescents with high parental income were more likely to fall short of physical activity guidelines (OR children: 1.30, 95% CI: 0.48–3.55; OR adolescents: 5.0, 95% CI: 0.66–37.6) compared to those with low parental income	Access to digital devices and entertainment options that can potentially reduce physical activity levels. However, the finding may be influenced by measurement errors in physical activity data	6

### 3.5. Parental socioeconomic status and fruits/vegetables

#### 3.5.1. Parental socioeconomic status and fruits/vegetables among children

Fourteen studies (48, 53, 79, 81, 82, 84, 89, 90, 95, 99, 106, 110, 111, 118) were identified that examined the association between SES and fruit and vegetable consumption. Ten of these studies found a positive link between high SES and children’s consumption of fruits and vegetables (48, 53, 82, 84, 95, 99, 106, 110, 111, 118), which implies that children from a high SES background had a higher chance of consuming high amounts of fruits and vegetables compared to those from low SES backgrounds.

#### 3.5.2. Parental socioeconomic status and fruits/vegetables among adolescents

From 14 studies that examined the association between SES and the consumption of fruits and vegetables, four studies found a positive association between high SES and the consumption of fruit and vegetables in adolescents (79, 81, 89, 90). A study by Vereecken et al. (82) reported that adolescents from Europe (West, South, North, Central, East), North America, and Asia with low SES were less likely to consume fruits and vegetables than those from high-SES backgrounds. Adolescents from low- SES families in Western Europe, Northern Europe, Southern Europe, North-Eastern Europe (except Estonia, Hungary, Latvia, Russia, and Ukraine), North America, and Asia were less likely to consume vegetables than their counterparts. Overall, high SES was associated with greater rates of fruit and vegetable consumption among children (10 studies or 71.42%) and adolescents (4 studies or 28.57%) compared to low SES (see Table 4).

**Table 4:** Studies examining the association between SES and fruits and vegetables consumption.

SN	Authors	Methodology description						Findings	Key strengths and limitations	Quality score
		Study design	Sample size (n)	Continent/country	Age range	SES measure	Method of analysis			
1	Mikki et al. (2010)	Cross-sectional survey	2,952	Palestine	13–15 years	Parental education	The multivariate linear regression model	Adolescents with high parental education consumed fruits and vegetables more frequently (6.3 times per week) than those with low parental education.	Healthy eating habits are more prone in highly educated families which can influence their children’s healthy dietary choices. One potential constraint of this study is the utilization of self-reported responses, which has the potential to impact both the validity and reliability of the findings.	8
2	Moore and Littlecott (2015)	Cross-sectional	9,194	Wales	11–16 years	SES	Mixed-effects logistic regression models	Children and adolescents with low SES had a lower likelihood (OR: 1.15, 95% CI: 1.10–1.20) of consuming fruits and vegetables compared to those from high SES.	Food insecurity can result in less frequent consumption of fruits and vegetables. However, this cross-sectional study lacks to established causal association between high SES and the consumption of fruits and vegetables.	8
3	Yannakoulia et al. (2016)	Cross-sectional, National representative	11,717	Greece	3–18 years	SES	Classification–regression tree analysis (CART) model	Children and adolescents from low SES consumed fewer fruits (31.2 and 25.3%, respectively) and vegetables (56.7 and 55.5%, respectively) than those from high SES.	Limited financial resources can make it difficult to afford fresh fruits and vegetables regularly. However, a low response rate might distort the findings	8
4	Richter et al. (2009a)	Cross-national survey	97,721	Europe (*n* = 30), Asia (*n* = 1), North America (*n* = 2)	13–15 years	SES	The multilevel logistic regression model	Adolescents with low SES had lower odds of consuming fruits and vegetables (OR boys: 0.61, 95% CI: 0.54–0.70; OR girls: 0.78, 95% CI: 0.73–0.84) than those with high SES.	Affordable food sources limit access to these nutritious options. However, self-reported adolescent behavioral patterns can make it difficult to estimate the inclusive results.	8
5	Richter et al. (2009)	Cross-national survey	86,667	Europe (*n* = 26) North America (*n* = 2)	11–15 years	SES	Logistic regression	Children and adolescents from various regions, including Southern, Northern, Western, Eastern, Central Europe, Northern America (USA and Canada), and Asia (Israel), had a lower likelihood of consuming vegetables if they were from low SES compared to high SES	SES disparities exist in many regions that can limit their ability to access and afford fresh vegetables. However, methodological constraints limit findings.	8
6	Svastisalee et al. (2012)	Cross-sectional	6,034	Denmark	11–15 years	Parental occupation	The multilevel logistic regression model	Children and adolescents from higher parental social class had a higher likelihood of consuming fruits (OR: 1.46, 95% CI: 1.24–1.71) and vegetables (OR: 1.86, 95% CI: 1.58–2.19) compared to those from lower social class	Higher social classes may be part of social circles that emphasize fruits and vegetables. Due to the nature of the data, this study is unable to draw a causality between SES and consumption of fruit and vegetables.	8
7	Vereecke et al. (2005)	Cross-national	114,558	28 European countries	11–15 years	SES, parental occupation	Multiple regression model	Children and adolescents from high parental SES had a higher likelihood of daily fruit consumption (OR: 1.530, 95% CI: 1.479–1.581) compared to those from low SES. Similarly, adolescents from high parental occupation levels had a higher likelihood of daily fruit consumption (OR: 1.186, 95% CI: 1.148–1.227) compared to their peers.	Healthy lifestyle prioritization contributes to daily fruit consumption. However, due to the poor classification of data measurement draws a poor conclusion.	6
8	Zaborskis et al. (2021)	Cross-national	192,755	42 countries including 40 countries from Europe, Canada, and Israel	11–15 years	SES	Logistic regression model and structural equation model	Adolescents with low parental SES were less likely to consume daily fruits (OR: 0.51; 95% CI: 0.49–0.53), vegetables (OR: 0.58; 95% CI: 0.56–0.60), and sweets (OR: 0.94; 95% CI: 0.90–0.97) than those with higher parental SES.	Access to affordable food and nutritional knowledge incorporated into diets. However, the findings are concerned with potential bias.	7
9	Voráčová et al. (2016)	Cross-sectional	10,831	Czech republic	11–15 years	SES	Logistic regression	Children and adolescents from higher parental SES backgrounds had higher odds of daily fruit (OR: 1.67, 95% CI: 1.34–2.08) and vegetable consumption (OR: 1.54, 95% CI: 1.22–2.00) compared to those from lower SES.	Food accessibility seems to increase the consumption of fruits and vegetables. However, eating habits as reported may have been influenced by social desirability	8
10	de Buhr and Tannen (2020)	Cross-sectional	4,294	German	6–13 years	SES	Spearman’s Rho correlations	Children from higher parental SES consumed more vegetables and salad (r: 0.100, 95% CI: 0.047–0.153) and fruits (r: 0.086, 95% CI: 0.032–0.139) than those from lower SES.	Financial resources, nutritional knowledge, and healthy lifestyle prioritization contribute to increased consumption of healthy food. However, it does not explain whether SES influences dietary choices or if dietary choices influence SES	8
11	Johansen et al. (2006)	Cross-sectional	3,458	Denmark	14–16 years	Maternal occupation	GEE logistic regression	Adolescents with high maternal occupation significantly consumed more fruits and vegetables (OR: 1.65, 95% CI: 1.15–2.36) compared to their peers.	High-occupation may cultivate a health-conscious environment that encourages the consumption of fruits and vegetables. However, the study failed to establish a causal association between SES and healthy food behavior.	6
12	Nardone et al. (2020)	Cross-sectional	58,976	Italy	11–15 years	Parental income and parental education	Logistic regression	Children and adolescents with high parental education were more likely to consume fruits and vegetables (OR: 0.54, 95% CI: 0.48–0.61), as were those with high parental income (OR: 0.70, 95% CI: 0.64–0.77) compared to their counterparts with lower education and income.	Health-conscious behavior may prioritize providing nutritious foods. The poor study setting did not provide a valid conclusion.	6
13	Al Sabbah et al. (2007)	Cross-sectional	8,885	Palestine	12–18 years	Maternal education	Logistic regression	Adolescents with high maternal education were more likely to consume more fruits (OR: 1.35, 95% CI: 1.19–1.53) and vegetables (OR: 1.10, 95% CI: 0.98–1.24) compared to those with low maternal education.	Nutritional information associated with consuming fruits and vegetables. Nevertheless, self-reported measurements might result in either an overestimation or underestimation of the findings.	6
14	Zaborskis et al. (2012)	Cross-sectional	33,230	Lithuania	11–15 years	Parental income	Binary logistic regression	Adolescents with high parental income were more likely to consume fruits daily (OR: 2.15, 95% CI: 1.86–2.49) and vegetables (OR: 1.12, 95% CI: 0.97–1.28) compared to those with low parental income.	Families with higher incomes often have better access to fresh fruits and vegetables on a daily basis. However, it is hard to make causality inferences due to the study design.	8

### 3.6. Parental socioeconomic status and dietary habits

#### 3.6.1. Parental socioeconomic status and dietary habits among children

A total of 46 studies were included in this review study. Of these, 18 studies (25, 48, 79, 81, 82, 89, 90, 97, 99, 104–106, 111, 112, 114, 116–118) examined the association between SES and healthy diet habits in childhood and adolescence. Of a total of 18 studies, six studies revealed a positive association between high SES and healthy diet habits (e.g., animal products, nutritious food, balanced diet, breakfast) in children (25, 48, 104, 111, 116, 117). Consequently, another four studies reported a positive association between high SES and unhealthy diet (e.g., Biscuits, pastries, irregular breakfast, sweet foods, and soft drinks) (82, 89, 106, 114), while three studies found a negative association between high SES and low consumption of unhealthy dietary foods (99, 105, 118).

#### 3.6.2. Parental socioeconomic status and dietary habits among adolescents

Out of 18 studies that examined the association between SES and dietary habits, five studies reported a positive association between high SES and consumption of healthy dietary food in adolescents (79, 81, 90, 97, 112), implying that adolescents with high parental SES had a higher probability of consuming a high proportion of dairy products, a regular breakfast, a healthy or nutritious diet, a balanced diet, and a low proportion of high-fat diet than those with low SES (see Table 5). In summary, six (33.33%) and five studies (27.77%) were positively associated with high SES and healthy dietary habits in childhood and adolescence, respectively.

**Table 5:** Studies examining the association between parental SES and diet habits.

SN	Authors	Methodology description						Findings	Key strengths and limitations	Quality score
		Study design	Sample size (n)	Continent/country	Age range	SES measure	Method of analysis			
1	Mikki et al. (2010)	Cross-sectional survey	2,952	Palestine	13–15 years	Parental education	The multivariate linear regression model	Adolescents with higher parental education were more likely to have a healthier dietary pattern compared to those with lower parental education	Awareness of health risks associated with poor dietary choices. This awareness can motivate them to emphasize healthy eating in their households. One potential constraint of this study is the utilization of self-reported responses, which has the potential to impact both the validity and reliability of the findings.	8
2	Yannakoulia et al. (2016)	Cross-sectional, National representative	11,717	Greece	3–18 years	SES	Classification–regression tree analysis (CART) model	Children and adolescents from low SES backgrounds had lower consumption of dairy products and daily breakfast than those from high SES backgrounds. Similarly, low SES adolescents had lower consumption of dairy products and daily breakfast than their high SES peers.	Lower-educated parents may have less knowledge about the nutritional benefits. So, they may not prioritize healthy foods in their children’s diets. However, self-reported adolescent behavioral patterns can make it difficult to estimate the inclusive results.	8
3	Richter et al. (2009a)	Cross-national survey	97,721	Europe (*n* = 30), Asia (*n* = 1), North America (*n* = 2)	13–15 years	SES	The multilevel logistic regression model	Adolescents with low SES had a reduced likelihood of consuming breakfast (OR boys: 0.75, 95% CI: 0.66–0.86; OR girls: 0.83, 95% CI: 0.75–0.93) compared to those with high SES	Economic constraints limit their access to a variety of nutritious breakfast options. However, the findings were influenced by the way physical activity is measured or reported leading to unusual results.	8
4	Poulain et al. (2019)	Longitudinal	1,223	German	3–18 years	SES	Mixed-effect models	Children (β: 0.05, 95% CI −0.03 to 0.13) and adolescents (β: 0.10, 95% CI: 0.01–0.18) in higher-SES were more likely to consume nutritious food compared to those in lower-SES families	Education and nutrition knowledge emphasize the importance of fruits, vegetables, and other healthy food items. However, this study may have limited representativeness across socioeconomic classes, potentially limiting its broader applicability.	9
5	Vereecke et al. (2005)	Cross-national	114,558	28 European countries	11–15 years	SES	Multiple regression model	Children and adolescents with high SES were more likely to consume a higher number of soft drinks (OR: 1.257, 95% CI: 1.211–1.305) compared to their peers.	Higher disposable may have greater purchasing power for non-essential items. Due to the poor classification of data measurement draws a poor conclusion.	6
6	Sinai et al. (2021)	Cross-sectional	3,902	Isreal	11–18 years	SES	Multiple regression model	Adolescents with higher SES had a higher consumption of plant-based foods, cereals, milk, and spreads (OR Plant-based food: 1.50, 95% CI: 1.23–1.82, OR Cereals and milk: 1.10, 95% CI: 0.91–1.33) compared to those with lower SES.	Access to resources and health consciousness make easier for people from high SES backgrounds to incorporate these foods into their diets. However, self-reported information could influence the results.	8
7	Yang (2021)	Longitudinal	1,040	South Korea	10–11 years	SES	Multiple linear regression model	Children from higher parental SES levels were more likely to consume healthy food (β = 0.07, *p* = 0.018) compared to their peers.	Parents prioritize healthy eating behaviors which are more likely to adopt similar habits to their children. However, this study limits the causal inferences	10
8	Esquius et al. (2021)	Cross-sectional	7,319	Spain	12–18 years	SES	Multilevel Poisson regression models	Adolescents with low SES were at a higher risk of skipping breakfast (PR boys: 1.28, 95% CI: 1.04–1.58; PR girls: 1.30, 95% CI: 1.12–1.52) compared to their peers	Time and routine constraints can make it difficult to prioritize breakfast. Self-reported adolescent diet patterns can be difficult to assess because of social desirability bias.	8
9	Park and Hwang (2017)	Cross-sectional	72,435	South-Korea	13–18 years	SES	Multivariate logistic regression	The odds of skipping breakfast were significantly higher (OR: 1.433, 95% CI: 1.347–1.523) for adolescents with low parental SES compared to those with high parental SES.	Food insecurity can result in irregular meal patterns, including breakfast-skipping. Nevertheless, there are certain methodological limitations in the study	8
10	Voráčová et al. (2016)	Cross-sectional	10,831	Czech Republic	11–15 years	SES	Logistic regression	Children and adolescents from higher parental SES backgrounds had lower odds of consuming sweets (OR: 0.79, 95% CI: 0.69–0.90) and soft drinks (OR: 0.41, 95% CI: 0.31–0.53) compared to their peers.	Parental monitoring can lead to healthier food choices for their children. However, eating habits as reported may have been influenced by social desirability bias.	8
11	Johansen et al. (2006)	Cross-sectional	3,458	Denmark	14–16 years	Maternal occupation	GEE logistic regression	Adolescents with unemployed mothers had significantly higher odds of irregular breakfast consumption (OR: 1.56, 95% CI: 1.06–2.29) compared to their peers.	Unemployment can lead to food insecurity in the household. In such cases, adolescents may skip meals, including breakfast. However, the study did not provide a clear pattern of how SES contributes to eating a healthy diet.	6
12	Morgan et al. (2021)	Longitudinal	176,094	Wales	11–16 years	SES	Multinomial logistic regression	Children and adolescents with low SES had a lower likelihood of daily sugar-sweet beverage consumption (RRR: 0.68, 95% CI: 0.66–0.70) and lower consumption of energy drinks (RRR: 0.67, 95% CI: 0.63–0.70) compared to those with high SES.	Low SES neighborhoods may have limited access to stores that offer soft drink beverage options. Methodological constraints affect the findings of this study.	8
13	Nardone et al. (2020)	Cross-sectional	58,976	Italy	11–15 years	Parental income and parental education	Logistic regression	Children and adolescents with higher parental education were less likely to skip breakfast (OR: 0.75, 95% CI: 0.67–0.84), as were those with higher parental income (OR: 0.84, 95% CI: 0.76–0.92).	Health consciousness can provide guidance on healthy eating habits, including breakfast. The poor study setting did not provide a valid conclusion.	6
14	Simetin et al. (2011)	Cross-sectional	3,296	Croatia	11–15 years	SES	Binary logistic regression	Children from high parental SES backgrounds had a higher likelihood of consuming regular breakfast (OR: 1.3, 95% CI: 1–1.8) compared to those from low parental SES.	Higher resources and knowledge emphasis on regular breakfast consumption. However, self-reporting bias and lack of causation limit conclusions on the high SES and consumption of diet.	6
15	Al Sabbah et al. (2007)	Cross-sectional	8,885	Palestine	12–18 years	Maternal education	Logistic regression	Adolescents with higher maternal education were more likely to consume sweets (OR: 1.15, 95% CI: 1.01–1.30) and soft drinks (OR: 1.28, 95% CI: 1.11–1.48) compared to those with low maternal education.	Consumption of sweets and soft drinks can be influenced by cultural and social norms. Nevertheless, self-reported measurements might result in either an overestimation or underestimation of the findings.	6
16	Lazzeri et al. (2014)	Cross-sectional	3,291	Italy	11–15 years	Parental income	Logistic regression	Children and adolescents with high parental income were more likely to have irregular breakfast consumption (OR children: 1.12, 95% CI: 0.60–2.09; OR adolescents: 1.80, 95% CI: 0.98–3.31) compared to those with low parental income.	Parents from high SES backgrounds may have demanding jobs or work long hours, which can affect the time allocated for breakfast preparation and consumption. However, the finding may be influenced by measurement errors.	6
17	Zaborskis et al. (2012)	Cross-sectional	33,230	Lithuania	11–15 years	Parental income	Binary logistic regression	Children and adolescents with high parental income were more likely to regularly consume sweets and chocolates, regularly drink soft drinks, and regularly consume biscuits and pastries (OR for sweets and chocolates: 1.48, 95% CI: 1.31–1.68; OR for soft drinks: 1.39, 95% CI: 1.21–1.60; OR for biscuits and pastries: 1.38, 95% CI: 1.17–1.63) compared to those with low parental income	Accessibility and parental time constraints can lead to an increase in the consumption of fast food including chocolates, soft drinks, biscuits, and pastries. However, it is hard to make causality inferences due to the study design.	8
18	Zaborskis et al. (2021)	Cross-national	192,755	42 countries including 40 countries from Europe, Canada, and Israel	11–15 years	SES	Logistic regression model and structural equation model	Children and adolescents from low parental SES were more likely to consume more soft drinks (OR: 1.25; 95% CI: 1.20, 1.30) than those from higher parental SES.	Soft drinks are often more affordable than healthier beverage options. However, the findings are concerned with potential bias.	7

### 3.7. Parental socioeconomic status and cannabis, marijuana, and illicit drug use by adolescents

In this literature review, we assessed three studies (47, 101, 103) out of 46 that examined the relationship between SES and cannabis use and illicit drugs. Of these, one study reported a negative association between high SES and cannabis use (47), while another found a negative relationship between high SES and marijuana consumption (101). Similarly, two other studies found a negative association between high SES and illicit drugs used by adolescents (101, 103). This indicates that adolescents with low SES were found to consume cannabis, marijuana, and illicit drugs more frequently than adolescents from high SES backgrounds. In summary, 66.66%, or two studies, reported a negative association between high SES and illicit drug used in adolescents (see Table 6).

**Table 6:** Studies examining the association between SES and cannabis, and illicit drug use.

SN	Authors	Methodology description						Findings	Key strengths and limitations	Quality score
		Study design	Sample size (n)	Continent/country	Age range	SES measure	Methodology			
1	Simetin et al. (2013)	Cross-sectional	1,601	Croatia	15 years	SES	Multi-level logistic regression	Adolescents from high SES backgrounds had a higher likelihood of cannabis consumption (OR: 1.49; SE: 0.22) compared to those from low SES.	High-SES adolescents have more disposable income, making cannabis and other drugs easier to afford. However, the association between socioeconomic factors and risk behaviors may be influenced by adolescents’ relative resilience to socioeconomic inequalities	8
2	Doku et al. (2012)	Cross-sectional	1,195	Ghana	12–18 years	SES	Logistic regression	Adolescents from low parental SES were more likely to use marijuana (OR: 12.4, 95% CI: 3.7–41.0) and illicit drugs (OR: 15.9, 95% CI: 3.7–67.8) than those from high parental SES.	Economic pressures can increase teenage stress and anxiety. They may be more tempted to utilize drugs. However, data was collected using self-report measures and utilized a cross-sectional research design. So, it cannot establish a causal association and there might be a chance of biases in the findings.	7
3	Lee et al. (2018)	Longitudinal	3,395	USA	12–16 years	Parental education		Adolescents with lower parental education were more likely to use illicit drugs (β: 0.08, 95% CI: 0.004–0.158) compared to those with higher parental education	Inequality in information and peer pressure enhance illicit drug use. However, the findings were distorted due to the measurement bias.	9

## 4. Discussion

Our study produced evidence, mostly from cross-sectional and longitudinal studies, that consistently demonstrates a strong relationship between SES and health behaviors. SES plays a major and well-documented influence in the onset and progression of chronic diseases in children and adolescents. Lower-income children and adolescents may have less access to frequent check-ups, preventative care, and early disease identification, increasing their risk of acquiring chronic disorders. Furthermore, SES influences the availability and cost of healthy food. Low-income families may struggle to offer adequate diets, which can contribute to poor eating habits, obesity, and linked chronic illnesses such as type 2 diabetes (52, 119, 120). Childhood and adolescence are regarded as pivotal stages in the development of life foundations in the general population. Focusing on younger ages is important because health behaviors can be learned and consolidated during these ages, affecting an individual’s health for the rest of their life. Therefore, this review study aims to examine the association between SES and health behaviors in children and adolescents.

The findings of this review demonstrate that children and adolescents from low SES backgrounds are more likely to engaged in unhealthy behaviors, such as smoking, alcohol consumption, physical inactivity, poor dietary choices, and drug use, when compared to their peers from higher SES backgrounds. These disparities in multiple health behaviors among children and adolescents may be possibly due to low parental SES, which limits access to healthy food, physical activity, and health education. Lower parental SES also correlates with low health literacy, which may lead to a lack of awareness about the importance of healthy behaviors. These findings align with existing literature, highlighting the impact of economic constraints in low-SES households, hindering access to health-promoting resources. Limited financial resources often result in inadequate nutrition, reduced physical activity opportunities, and lower educational attainment, contributing to reduced health awareness (120–124). This knowledge and resource gap influences healthy behaviors in individuals, children, and adolescents. As a result, it is critical to emphasize the need for tailored interventions for disadvantaged children and adolescents.

In the context of SES and smoking behavior among children and adolescents, the results of this review study reported that children and adolescents with low parental SES backgrounds had heightened vulnerability to early exposure to smoking during childhood and early initiation of smoking during adolescence. This susceptibility may be attributed to a confluence of factors, including pervasive tobacco advertising, normalization of smoking within their social environments, easy access to cigarettes, limited social support for smoking cessation, heightened nicotine dependence, and the burden of stressful life circumstances. These findings substantiate the existing body of literature, which consistently demonstrates an elevated prevalence of smoking within lower SES strata, often attributed to factors such as a paucity of robust social support networks and the presence of stress-inducing lifestyles. Furthermore, children from low SES backgrounds are approximately 6.6 times more likely to be exposed to secondhand smoke within their parental residences compared to their counterparts in high- SES households (125–127). This discrepancy underscores the urgent need for targeted interventions to mitigate the adverse consequences of smoking in vulnerable populations, particularly during the critical period of life.

On the other hand, alcohol consumption was significantly higher among adolescents with high SES. These findings align with those of previous research, reinforcing the robust association between SES and adolescent alcohol consumption. Specifically, adolescents from high parental SES backgrounds exhibit increased odds of alcohol use (OR = 1.4, 95% CI = 1.19–1.78) (128). Recent research by Torchyan et al. (129) further substantiates this phenomenon, indicating a heightened likelihood of weekly alcohol consumption (OR = 1.24, 95% CI = 1.16–1.32) among high-SES adolescents when compared to their counterparts (129). This persistent pattern has been confirmed in other studies, indicating that high-SES adolescents are more likely to consume alcohol under parental supervision than their counterparts, (130). This could be explained through social and cultural activities (business meetings, and, party celebrations), the availability of alcohol at home, and the availability of pocket money to purchase alcohol (131). This review underlines early alcohol initiation among children with high SES, which is often influenced by parental drinking behavior (132). Thus, parental discretion in alcohol consumption around children is pivotal in preventing negative alcohol-related behavior (133). Therefore, this study suggests that parents should take care while drinking alcohol in front of their children to prevent them from developing bad alcohol-related behavior.

In addition, our review study found that children and adolescents with high SES were more likely to be engaged in physical exercise. These findings are consistent with previous studies and led to the conclusion that parental SES (OR = 2.73, 95% CI = 2.18, 3.42) were significantly (*p* < 0.05) associated with participation in indoor and outdoor physical activities by children and adolescents (134, 135). If parents had a good family income, there would be a higher chance of availability of goods and material resources at home (136). This availability of materials would make it possible to be more engaged in physical activity rather than spending more time watching television or being inactive (137). However, children and adolescents from lower parental SES backgrounds were found to participate less often in physical activity but were more often involved in sedentary behaviors. Previous studies have found that poor parental SES backgrounds were significantly associated with poor physical activity, high sedentary activity, and high screen time in children and adolescents (113, 138, 139). Moreover, there was ample evidence to suggest that more children and adolescents from deprived SES were more likely to spend more time inside the home due to a lack of a secure neighborhood, lack of green areas for sports and recreational activities, and the cost associated with physical activity (140, 141).

In relation to SES and diet (e.g., dairy products, fruit, vegetables, breakfast, soft drinks, and high-fat diet), we found that higher SES was positively associated with the consumption of breakfast, dairy products, fruit, vegetables, and a balanced diet, but negatively associated with consumption of sugar, sugar items, high- fat diet, and soft drinks. The findings of this review study are consistent with those of other studies that show that parental SES has a strong influence on children’s diet (142). These findings show that children and adolescents from high-SES families consume a healthy proportion of calories (*β* = 1.86, SE = 0.76) rather than high-energy foods (143). Children and adolescents from low-SES families may have few food options and may even face food insecurity and scarcity at home (144). Therefore, low parental SES may lead to low price food, high-fat diet, high- salt food, energy-dense food, and low intake of regular breakfast, dairy products, fruits, and vegetables during childhood and adolescence (144–147).

Moreover, in the context of SES and cannabis and illicit drug abuse in adolescents, there was a correlation between the consumption of cannabis and illicit drug abuse and low parental SES, poor schooling, unsafe neighborhoods, and stressful daily life events. Thus, it is important to consider how parental SES particularly affects an adolescent’s health behaviors. This review study revealed that 66.66% of the adolescents had consumed illicit drugs. These conditions increase the risk of physical, psychological, social, and emotional competence in adolescents. These findings could help to provide information about how low parental SES affects children and adolescents, and thus motivate authorities to carry out preventative activities and appropriate rules, regulations, and policies to promote healthy lifestyles in children and adolescents (148). Overall, risky health behaviors were found to be a major concern, particularly in children and adolescents with low parental SES. These conditions may increase the risk of poor health and development in childhood and adolescence. Therefore, to control these issues, an appropriate strategy helps to protect and prevent risky health behaviors in children and adolescents and gives them equal rights, services, and facilities to fight against inequalities. Hence, this study indicated that parental SES play a significant role in developing social and emotional competence and positive health outcomes in children and adolescents. Therefore, authorities and government bodies should pay more attention to the health and health behaviors of every individual, including children and adolescents, and provide support to children and adolescents, especially those with low parental SES.

## 5. Strength and limitations

This study comprehensively examined the association between SES and health behaviors, including protecting and impairing health behaviors in children and adolescents across the world. Moreover, this study used multiple databases and followed a structural research process that provided transparent, unbiased, and reliable information on SES and health behaviors. In summary, this study’s strengths lie in its comprehensive approach, use of multiple databases, adherence to a structured research process, and commitment to providing unbiased information. However, this study had certain limitations. The heterogeneity of the studies makes Meta-Analysis not possible. Another limitation is no risk of bias and no registration in “PROSPERO,” was remedied by that two researchers have independently assessed each article, and that a Quality Score was provided (Supplementary Table S4) as well as a “PRISMA” approach (Supplementary Table S3).

## 6. Conclusion

The current study revealed a robust association between low parental SES and a myriad of unhealthy behaviors (high smoking behavior, low physical exercise, illicit drug use, low consumption of fruit and vegetables, and unhealthy diet) in children and adolescents. However, alcohol consumption was more common in adolescents with a high parental SES. Based on these significant findings, this study underscores the urgent need for an appropriate intervention program that caters to the unique needs of children and adolescents from low-SES families. By implementing measures such as free all-day schools with complementary meals, free after-school activities, and supervised homework sessions, we can bridge the gaps in opportunities, capabilities, and productivity, thereby fostering healthier and more promising futures for these individuals.

## Data availability statement

The original contributions presented in the study are included in the article/Supplementary material, further inquiries can be directed to the corresponding author.

## Author contributions

NG: conceptualization, data curation, methodology, software, formal analysis, and original drafting. GD: methodology, reviewing, editing, supervision, and validation. MR: reviewing, editing, supervision, and validation. RK: conceptualization, reviewing, editing, supervision, and validation. All authors contributed to the article and approved the submitted version.

## Abbreviations

JBI: Joanna Briggs Institute
PA: Physical Activity
PRISMA: Preferred Reporting for Systematic Review and Meta-analysis
SES: Socioeconomic Status
SUMARI: System for the Unified Management of the Assessment and Review of Information

## References

[1] Kivimäki M Batty GD Pentti J Shipley MJ Sipilä PN Nyberg ST . Association between socioeconomic status and the development of mental and physical health conditions in adulthood: a multi-cohort study. Lancet Public Health. (2020) 5:e140–9. doi: 10.1016/S2468-2667(19)30248-8, PMID: 32007134

[2] Yusuf S Joseph P Rangarajan S Islam S Mente A Hystad P . Modifiable risk factors, cardiovascular disease, and mortality in 155 722 individuals from 21 high-income, middle-income, and low-income countries (PURE): a prospective cohort study. Lancet. (2020) 395:795–808. doi: 10.1016/S0140-6736(19)32008-2, PMID: 31492503 PMC8006904

[3] Qin Z Wang N Ware RS Sha Y Xu F. Lifestyle-related behaviors and health-related quality of life among children and adolescents in China. Health Qual Life Outcomes. (2021) 19:8. doi: 10.1186/s12955-020-01657-w, PMID: 33407589 PMC7788787

[4] Macsinga I Nemeti I. The relation between explanatory style, locus of control and self-esteem in a sample of university students. Procedia Soc Behav Sci. (2012) 33:25–9. doi: 10.1016/j.sbspro.2012.01.076

[5] Janowski K Kurpas D Kusz J Mroczek B Jedynak T. Health-related behavior, profile of health locus of control and acceptance of illness in patients suffering from chronic somatic diseases. PLoS One. (2013) 8:e63920. doi: 10.1371/journal.pone.0063920, PMID: 23675516 PMC3651173

[6] Patterson RE Haines PS Popkin BM. Health lifestyle patterns of US adults. Prev Med. (1994) 23:453–60. doi: 10.1006/pmed.1994.1062 7971872

[7] Hirdes JP Forbes WF. The importance of social relationships, socioeconomic status and health practices with respect to mortality among healthy Ontario males. J Clin Epidemiol. (1992) 45:175–82. doi: 10.1016/0895-4356(92)90010-K, PMID: 1573434

[8] Wiley JA Camacho TC. Life-style and future health: evidence from the Alameda County study. Prev Med. (1980) 9:1–21. doi: 10.1016/0091-7435(80)90056-0, PMID: 7360725

[9] McGinnis JM Foege WH. Actual causes of death in the United States. JAMA. (1993) 270:2207. doi: 10.1001/jama.1993.03510180077038 8411605

[10] Braveman P Gottlieb L. The social determinants of health: it's time to consider the causes of the causes. Public Health Rep. (2014) 129:19–31. doi: 10.1177/00333549141291S2, PMID: 24385661 PMC3863696

[11] Peacock A Leung J Larney S Colledge S Hickman M Rehm J . Global statistics on alcohol, tobacco and illicit drug use: 2017 status report. Addiction. (2018) 113:1905–26. doi: 10.1111/add.14234, PMID: 29749059

[12] King DE Mainous AG III Carnemolla M Everett CJ. Adherence to healthy lifestyle habits in US adults, 1988-2006. Am J Med. (2009) 122:528–34. doi: 10.1016/j.amjmed.2008.11.013, PMID: 19486715

[13] Guthold R Stevens GA Riley LM Bull FC. Worldwide trends in insufficient physical activity from 2001 to 2016: a pooled analysis of 358 population-based surveys with 1· 9 million participants. Lancet Glob Health. (2018) 6:e1077–86. doi: 10.1016/S2214-109X(18)30357-7, PMID: 30193830

[14] Falci CD. Self-esteem and mastery trajectories in high school by social class and gender. Soc Sci Res. (2011) 40:586–601. doi: 10.1016/j.ssresearch.2010.12.013, PMID: 21423844 PMC3057090

[15] Twenge JM Campbell WK. Self-esteem and socioeconomic status: a meta-analytic review. Personal Soc Psychol Rev. (2002) 6:59–71. doi: 10.1207/S15327957PSPR0601_3

[16] Schwartz CE Zhang J Stucky BD Michael W Rapkin BD. Is the link between socioeconomic status and resilience mediated by reserve-building activities: mediation analysis of web-based cross-sectional data from chronic medical illness patient panels. BMJ Open. (2019) 9:e025602. doi: 10.1136/bmjopen-2018-025602, PMID: 31154302 PMC6549651

[17] Zou R Xu X Hong X Yuan J. Higher socioeconomic status predicts less risk of depression in adolescence: serial mediating roles of social support and optimism. Front Psychol. (2020) 11:1955. doi: 10.3389/fpsyg.2020.01955, PMID: 32849145 PMC7425112

[18] Conger RD Donnellan MB. An interactionist perspective on the socioeconomic context of human development. Annu Rev Psychol. (2007) 58:175–99. doi: 10.1146/annurev.psych.58.110405.085551, PMID: 16903807

[19] Quon EC McGrath JJ. Subjective socioeconomic status and adolescent health: a meta-analysis. Health Psychol. (2014) 33:433–47. doi: 10.1037/a0033716, PMID: 24245837 PMC5756083

[20] Pechey R Monsivais P. Socioeconomic inequalities in the healthiness of food choices: exploring the contributions of food expenditures. Prev Med. (2016) 88:203–9. doi: 10.1016/j.ypmed.2016.04.012, PMID: 27095324 PMC4910945

[21] Okamoto S. Parental socioeconomic status and adolescent health in Japan. Sci Rep. (2021) 11:12089. doi: 10.1038/s41598-021-91715-0 34103647 PMC8187727

[22] Chen Q Kong Y Gao W Mo L. Effects of socioeconomic status, parent–child relationship, and learning motivation on reading ability. Front Psychol. (2018) 9:1297. doi: 10.3389/fpsyg.2018.01297 30090082 PMC6068389

[23] Charonis A Kyriopoulos I-I Spanakis M Zavras D Athanasakis K Pavi E . Subjective social status, social network and health disparities: empirical evidence from Greece. Int J Equity Health. (2017) 16:40. doi: 10.1186/s12939-017-0533-y, PMID: 28241834 PMC5327516

[24] Belsky J Bell B Bradley RH Stallard N Stewart-Brown SL. Socioeconomic risk, parenting during the preschool years and child health age 6 years. Eur J Pub Health. (2007) 17:508. doi: 10.1093/eurpub/ckl261 17170020

[25] Poulain T Vogel M Sobek C Hilbert A Körner A Kiess W. Associations between socio-economic status and child health: findings of a large German cohort study. Int J Env Res Public Health. (2019) 16:677. doi: 10.3390/ijerph16050677 30813530 PMC6427670

[26] Hasan E. Inequalities in health care utilization for common illnesses among under five children in Bangladesh. BMC Pediatr. (2020) 20:192. doi: 10.1186/s12887-020-02109-6, PMID: 32366236 PMC7197176

[27] Li J Wang J Li JY Qian S Jia RX Wang YQ . How do socioeconomic status relate to social relationships among adolescents: a school-based study in East China. BMC Pediatr. (2020) 20:271. doi: 10.1186/s12887-020-02175-w, PMID: 32493261 PMC7268251

[28] Shaw BA Agahi N Krause N. Are changes in financial strain associated with changes in alcohol use and smoking among older adults? J Stud Alcohol Drugs. (2011) 72:917–25. doi: 10.15288/jsad.2011.72.917, PMID: 22051205 PMC3211962

[29] Kipping RR Smith M Heron J Hickman M Campbell R. Multiple risk behaviour in adolescence and socio-economic status: findings from a UK birth cohort. Eur J Pub Health. (2015) 25:44–9. doi: 10.1093/eurpub/cku078, PMID: 24963150 PMC4304374

[30] Umeda M Oshio T Fujii M. The impact of the experience of childhood poverty on adult health-risk behaviors in Japan: a mediation analysis. Int J Env Res Public Health. (2015) 14:145. doi: 10.1186/s12939-015-0278-4, PMID: 26645322 PMC4673773

[31] Gong WJ Fong DYT Wang MP Lam TH Chung TWH Ho SY. Increasing socioeconomic disparities in sedentary behaviors in Chinese children. BMC Public Health. (2019) 19:754. doi: 10.1186/s12889-019-7092-7, PMID: 31196044 PMC6567653

[32] Assari S Caldwell CH Mincy R. Family socioeconomic status at birth and youth impulsivity at age 15; blacks’ diminished return. Children. (2018) 5:58. doi: 10.3390/children5050058 29724004 PMC5977040

[33] Assari S Smith J Mistry R Farokhnia M Bazargan M. Substance use among economically disadvantaged African American older adults; objective and subjective socioeconomic status. Int J Environ Res Public Health. (2019) 16:1826. doi: 10.3390/ijerph16101826, PMID: 31126049 PMC6572418

[34] Ben-Shlomo Y Kuh D. A life course approach to chronic disease epidemiology: conceptual models, empirical challenges and interdisciplinary perspectives. Int J Epidemiol. (2002) 31:285–93. doi: 10.1093/ije/31.2.285, PMID: 11980781

[35] Due P Krølner R Rasmussen M Andersen A Trab Damsgaard M Graham H . Pathways and mechanisms in adolescence contribute to adult health inequalities. Scand J Public Health. (2011) 39:62. doi: 10.1177/14034948103959 21382850

[36] United Nations International Children’s Emergency Fund. The state of the world’s children, growing well in a changing world children, food and nutrition. (2019). Available at: https://www.unicef.org/media/60806/file/SOWC-2019.pdf (Accessed November 15, 2021).

[37] United Nation. Peace, dignity and equality on a healthy planet. (2019). Available at: https://www.un.org/en/global-issues/children (Accessed October 25, 2021).

[38] Reiss F. Socioeconomic inequalities and mental health problems in children and adolescents: a systematic review. Soc Sci Med. (2013) 90:24–31. doi: 10.1016/j.socscimed.2013.04.026, PMID: 23746605

[39] Ross CE Mirowsky J. The interaction of personal and parental education on health. Soc Sci Med. (2011) 72:591. doi: 10.1016/j.socscimed.2010.11.028 21227556 PMC3049298

[40] Simkiss D. Inequalities in children’s health in the UK. Paediatr Child Health. (2014) 24:103.

[41] Hahn RA Truman BI. Education improves public health and promotes health equity. Int J Health Serv. (2015) 45:657–78. doi: 10.1177/002073141558598, PMID: 25995305 PMC4691207

[42] Pearce A Mason K Fleming K Taylor-Robinson D Whitehead M. Reducing inequities in health across the life-course: early years, childhood and adolescence. WHO Regional Office for Europe: Denmark (2020).

[43] Baker A Bentley C Carr D Connolly AM Heasman M Johnson C . Reducing health inequalities: system, scale and sustainability. London: Public Health England (2017).

[44] World Health Organization. Situation of child and adolescent health in Europe. Copenhagen, Denmark: WHO Regional Office for Europe (2018).

[45] Chen E Matthews KA Boyce WT. Socioeconomic differences in children's health: how and why do these relationships change with age? Psychol Bull. (2002) 128:295–329. doi: 10.1037/0033-2909.128.2.295, PMID: 11931521

[46] Hanson MD Chen E. Socioeconomic status and health behaviors in adolescence: a review of the literature. J Behav Med. (2007) 30:263–85. doi: 10.1007/s10865-007-9098-3 17514418

[47] Simetin IP Kern J Kuzman M Pfortner TK. Inequalities in Croatian pupils’ risk behaviors associated to socioeconomic environment at school and area level: a multilevel approach. Soc Sci Med. (2013) 98:154–61. doi: 10.1016/j.socscimed.2013.09.021, PMID: 24331894

[48] Yannakoulia M Lykou A Kastorini CM Papasaranti ES Petralias A Veloudaki A . Socio-economic and lifestyle parameters associated with diet quality of children and adolescents using classification and regression tree analysis: the DIATROFI study. Public Health Nutr. (2016) 19:339–47. doi: 10.1017/S136898001500110X, PMID: 25892409 PMC10270971

[49] Liu Y Wang M Tynjälä J Villberg J Lv Y Kannas L. Socioeconomic differences in adolescents’ smoking: a comparison between Finland and Beijing, China. BMC Public Health. (2016) 16:805. doi: 10.1186/s12889-016-3476-0, PMID: 27534849 PMC4989516

[50] Melotti R Heron J Hickman M Macleod J Araya R Lewis G. Adolescent alcohol and tobacco use and early socioeconomic position: the ALSPAC birth cohort. Pediatrics. (2011) 127:e948–55. doi: 10.1542/peds.2009-3450, PMID: 21402626

[51] Krist L Bürger C Ströbele-Benschop N Roll S Lotz F Rieckmann N . Association of individual and neighbourhood socioeconomic status with physical activity and screen time in seventh-grade boys and girls in Berlin, Germany: a cross-sectional study. BMJ Open. (2017) 7:e017974. doi: 10.1136/bmjopen-2017-017974 PMC5770905 29288179

[52] Obita G Alkhatib A. Disparities in the prevalence of childhood obesity-related comorbidities: a systematic review. Front Public Health. (2022) 10:923744. doi: 10.3389/fpubh.2022.923744, PMID: 35874993 PMC9298527

[53] Moore GF Littlecott HJ. School-and family-level socioeconomic status and health behaviors: multilevel analysis of a national survey in Wales, United Kingdom. J Sch Health. (2015) 85:267. doi: 10.1111/josh.12242 25731201 PMC4368681

[54] Haddad MR Sarti FM. Sociodemographic determinants of health behaviors among Brazilian adolescents: trends in physical activity and food consumption, 2009–2015. Appetite. (2020) 144:104454. doi: 10.1016/j.appet.2019.104454, PMID: 31521768

[55] Currie C Zanotti C Morgan A Currie D De Looze M Roberts C . Social determinants of health and well-being among young people. Health behaviour in school-aged children (HBSC) study: international report from the 2009/2010 survey. (Health Policy for Children and Adolescents). Copenhagen, Denmark: WHO Regional Office for Europe (2012).

[56] Jackson CA Henderson M Frank JW Haw SJ. An overview of prevention of multiple risk behaviour in adolescence and young adulthood. J Public Health. (2012) 34:i31–40. doi: 10.1093/pubmed/fdr113 22363029

[57] McInnes MD Moher D Thombs BD McGrath TA Bossuyt PM Clifford T . Preferred reporting items for a systematic review and meta-analysis of diagnostic test accuracy studies: the PRISMA-DTA statement. JAMA. (2018) 319:388. doi: 10.1001/jama.2017.19163 29362800

[58] Moher D Liberati A Tetzlaff J Altman DG Group* P. Preferred reporting items for systematic reviews and meta-analyses: the PRISMA statement. Ann Intern Med. (2009) 151:264. doi: 10.7326/0003-4819-151-4-200908180-00135 19622511

[59] Moher D Shamseer L Clarke M Ghersi D Liberati A Petticrew M . Preferred reporting items for systematic review and meta-analysis protocols (PRISMA-P) 2015 statement. Syst Rev. (2015) 4:1. doi: 10.1186/2046-4053-4-1, PMID: 25554246 PMC4320440

[60] US Department of Health. Pediatric expertise for advisory panels - guidance for industry and FDA staff. Geretta Wood: US Department of Health and Human Services Food and Drug Administration, Center for Devices and Radiological Health (2003).

[61] Johns Hopkins Medicine. The growing child: adolescent 13 to 18 years. Available at: https://www.hopkinsmedicine.org/health/wellness-and-prevention/the-growing-child-adolescent-13-to-18-years (Accessed November 22, 2021).

[62] Munn Z Aromataris E Tufanaru C Stern C Porritt K Farrow J . The development of software to support multiple systematic review types: the Joanna Briggs institute system for the unified management, assessment and review of information (JBI SUMARI). Int J Evid Based Healthc. (2019) 17:36–43. doi: 10.1097/XEB.0000000000000152 30239357

[63] Moola S Munn Z Tufanaru C Aromataris E Sears K Sfetcu R. Chapter 7: Systematic reviews of etiology and risk In: Aromataris E Munn Z, editors. Joanna Briggs Institute Reviewer’s Manual: The Joanna Briggs Institute. (2017).

[64] Munn Z Moola S Lisy K Riitano D Tufanaru C. Methodological guidance for systematic reviews of observational epidemiological studies reporting prevalence and cumulative incidence data. JBI Evid. Implement. (2015) 13:147–53. doi: 10.1097/XEB.0000000000000054, PMID: 26317388

[65] Piper C. System for the unified management, assessment, and review of information (SUMARI). J Med Libr Assoc. (2019) 107:634. doi: 10.5195/jmla.2019.790

[66] Dijkshoorn AB van Stralen HE Sloots M Schagen SB Visser-Meily JM Schepers VP. Prevalence of cognitive impairment and change in patients with breast cancer: a systematic review of longitudinal studies. Psychooncol. (2021) 30:635–48. doi: 10.1002/pon.5623, PMID: 33533166 PMC8248098

[67] Hoy D Brooks P Woolf A Blyth F March L Bain C . Assessing risk of bias in prevalence studies: modification of an existing tool and evidence of interrater agreement. J Clin Epidemiol. (2012) 65:934. doi: 10.1016/j.jclinepi.2011.11.014 22742910

[68] Johnston M Johnston DW. Assessment and measurement issues. In comprehensive clinical psychology. Johnston M Johnston DW Bellack AS Hersen M, Eds. Elsevier Science Publishers B.V. (2001) 113–135.

[69] Arnett JJ. Emerging adulthood: a theory of development from the late teens through the twenties. Am Psychol. (2000) 55:469–80. doi: 10.1037/0003-066X.55.5.469, PMID: 10842426

[70] Konner M. The evolution of childhood: relationships, emotion, mind. Cambridge, MA: Harvard University Press (2011).

[71] Armstrong KH Ogg JA Sundman-Wheat AN St John Walsh A Armstrong KH Ogg JA . Early childhood development theories. New York, NY: Springer (2014).

[72] Woodhead M. Early childhood development: a question of rights. Int J Early Child. (2005) 37:79–98. doi: 10.1007/BF03168347

[73] Arnett JJ. Adolescent storm and stress, reconsidered. Am Psychol. (1999) 54:317–26. doi: 10.1037/0003-066X.54.5.317, PMID: 10354802

[74] Adler NE Epel ES Castellazzo G Ickovics JR. Relationship of subjective and objective social status with psychological and physiological functioning: preliminary data in healthy. White Women Health Psychol. (2000) 19:586–92. doi: 10.1037/0278-6133.19.6.586, PMID: 11129362

[75] Doshi T Smalls BL Williams JS Wolfman TE Egede LE. Socioeconomic status and cardiovascular risk control in adults with diabetes. Am J Med Sci. (2016) 352:36–44. doi: 10.1016/j.amjms.2016.03.020, PMID: 27432033 PMC4955859

[76] Bradley RH Corwyn RF. Socioeconomic status and child development. Annu Rev Psychol. (2002) 53:371–99. doi: 10.1146/annurev.psych.53.100901.135233 11752490

[77] Howe LD Galobardes B Matijasevich A Gordon D Johnston D Onwujekwe O . Measuring socio-economic position for epidemiological studies in low-and middle-income countries: a methods of measurement in epidemiology paper. Int J Epidemiol. (2012) 41:871. doi: 10.1093/ije/dys037 22438428 PMC3396323

[78] Bhusal M Gautam N Lim A Tongkumchum P. Factors associated with stillbirth among pregnant women in Nepal. J Prev Med Public Health. (2019) 52:154–60. doi: 10.3961/jpmph.18.270, PMID: 31163950 PMC6549008

[79] Johansen A Rasmussen S Madsen M. Health behaviour among adolescents in Denmark: influence of school class and individual risk factors. Scand J Public Health. (2006) 34:32–40. doi: 10.1080/14034940510032, PMID: 16449042

[80] Piko BF Fitzpatrick KM. Socioeconomic status, psychosocial health and health behaviours among Hungarian adolescents. Eur J Pub Health. (2007) 17:353–60. doi: 10.1093/eurpub/ckl257, PMID: 17130141

[81] Richter M Erhart M Vereecken CA Zambon A Boyce W Gabhainn SN. The role of behavioural factors in explaining socio-economic differences in adolescent health: a multilevel study in 33 countries. Soc Sci Med. (2009) 69:396. doi: 10.1016/j.socscimed.2009.05.023 19540029

[82] Vereecken CA Inchley J Subramanian SV Hublet A Maes L. The relative influence of individual and contextual socio-economic status on consumption of fruit and soft drinks among adolescents in Europe. Eur J Pub Health. (2005) 15:224–32. doi: 10.1093/eurpub/cki005, PMID: 15905182

[83] Richter M Leppin A Gabhainn SN. The relationship between parental socio-economic status and episodes of drunkenness among adolescents: findings from a cross-national survey. BMC Public Health. (2006) 6:289. doi: 10.1186/1471-2458-6-289, PMID: 17132161 PMC1693920

[84] Richter M Vereecken CA Boyce W Maes L Gabhainn SN Currie CE. Parental occupation, family affluence and adolescent health behaviour in 28 countries. Int J Public Health. (2009) 54:203. doi: 10.1007/s00038-009-8018-4 19347249

[85] Andersen A Holstein BE Due P. School-related risk factors for drunkenness among adolescents: risk factors differ between socio-economic groups. Eur J Pub Health. (2007) 17:27–32. doi: 10.1093/eurpub/ckl071, PMID: 16837514

[86] Kislitsyna O Stickley A Gilmore A McKee M. The social determinants of adolescent smoking in Russia in 2004. Int J Public Health. (2010) 55:619. doi: 10.1007/s00038-010-0196-6 20890629

[87] Doku D Koivusilta L Rainio S Rimpela A. Socioeconomic differences in smoking among Finnish adolescents from 1977 to 2007. J Adolesc Health. (2010) 47:479. doi: 10.1016/j.jadohealth.2010.03.012 20970083

[88] Doku D Koivusilta L Raisamo S Rimpela A. Do socioeconomic differences in tobacco use exist also in developing countries? A study of Ghanaian adolescents. BMC Public Health. (2010) 10:758. doi: 10.1186/1471-2458-10-758, PMID: 21143849 PMC3016387

[89] Al Sabbah H Vereecken C Kolsteren P Abdeen Z Maes L. Food habits and physical activity patterns among Palestinian adolescents: findings from the national study of Palestinian schoolchildren (HBSC-WBG2004). Public Health Nutr. (2007) 10:739–46. doi: 10.1017/S1368980007665501, PMID: 17381946

[90] Mikki N Abdul-Rahim HF Shi ZM Holmboe-Ottesen G. Dietary habits of Palestinian adolescents and associated sociodemographic characteristics in Ramallah, Nablus and Hebron governorates. Public Health Nutr. (2010) 13:1419–29. doi: 10.1017/S1368980010000662, PMID: 20441660

[91] Andersen A Holstein BE Due P. Large-scale alcohol use and socioeconomic position of origin: longitudinal study from ages 15 to 19 years. Scand J Public Health. (2008) 36:326. doi: 10.1177/14034948070869 18519304

[92] Pavon DJ Ortega FP Ruiz JR Romero VE Artero EG Urdiales DM . Socioeconomic status influences physical fitness in European adolescents independently of body fat and physical activity: the HELENA study. Nutr Hosp. (2010) 25:311. doi: 10.3305/nh.2010.25.2.4596 20449543

[93] Moor I Rathmann K Lenzi M Pförtner TK Nagelhout GE de Looze M . Socioeconomic inequalities in adolescent smoking across 35 countries: a multilevel analysis of the role of family, school and peers. Eur J Public Health. (2015) 25:457–63. doi: 10.1093/eurpub/cku244, PMID: 25713016

[94] Hankonen N Heino MT Kujala E Hynynen ST Absetz P Araújo-Soares V . What explains the socioeconomic status gap in activity? Educational differences in determinants of physical activity and screentime. BMC Public Health. (2017) 17:144. doi: 10.1186/s12889-016-3880-5, PMID: 28143461 PMC5286840

[95] Svastisalee CM Holstein BE Due P. Fruit and vegetable intake in adolescents: association with socioeconomic status and exposure to supermarkets and fast food outlets. J Nutr Metab. (2012) 2012:1–9. doi: 10.1155/2012/185484, PMID: 22988491 PMC3440941

[96] Levin KA Dundas R Miller M McCartney G. Socioeconomic and geographic inequalities in adolescent smoking: a multilevel cross-sectional study of 15 year olds in Scotland. Soc Sci Med. (2014) 107:162–70. doi: 10.1016/j.socscimed.2014.02.016, PMID: 24607678 PMC3988930

[97] Park MH Hwang EH. Effects of family affluence on the health behaviors of Korean adolescents. Jpn J Nurs Sci. (2017) 14:173. doi: 10.1111/jjns.12146 28670857

[98] Henriksen PW Rayce SB Melkevik O Due P Holstein BE. Social background, bullying, and physical inactivity: national study of 11- to 15-year-olds. Scand J Med Sci Sports. (2016) 26:1249–55. doi: 10.1111/sms.12574, PMID: 26454139

[99] Voráčová J Sigmund E Sigmundová D Kalman M. Family affluence and the eating habits of 11- to 15-year-old Czech adolescents: HBSC 2002 and 2014. Int J Environ Res Public Health. (2016) 13:1034. doi: 10.3390/ijerph13101034, PMID: 27783063 PMC5086773

[100] Melotti R Lewis G Hickman M Heron J Araya R Macleod J. Early life socio-economic position and later alcohol use: birth cohort study. Addiction. (2013) 108:516–25. doi: 10.1111/add.12018, PMID: 23164048 PMC4150526

[101] Doku D Koivusilta L Rimpela A. Socioeconomic differences in alcohol and drug use among Ghanaian adolescents. Addict Behav. (2012) 37:357. doi: 10.1016/j.addbeh.2011.11.020 22154504

[102] Liu Y Wang M Tynjala J Villberg J Lv Y Kannas L. Socioeconomic inequalities in alcohol use of adolescents: the differences between China and Finland. Int J Public Health. (2013) 58:177–85. doi: 10.1007/s00038-012-0432-3, PMID: 23188069

[103] Lee JO Cho J Yoon Y Bello MS Khoddam R Leventhal AM. Developmental pathways from parental socioeconomic status to adolescent substance use: alternative and complementary reinforcement. J Youth Adolesc. (2018) 47:334–48. doi: 10.1007/s10964-017-0790-5, PMID: 29188410 PMC5790622

[104] Simetin IP Kuzman M Franelic IP Pristas I Benjak T Dezeljin JD. Inequalities in Croatian pupils' unhealthy behaviours and health outcomes: role of school, peers and family affluence. Eur J Pub Health. (2011) 21:122–8. doi: 10.1093/eurpub/ckq002, PMID: 20159771

[105] Lazzeri G Azzolini E Pammolli A Simi R Meoni V Giacchi MV. Factors associated with unhealthy behaviours and health outcomes: a cross-sectional study among tuscan adolescents (Italy). Int J Equity Health. (2014) 13:83. doi: 10.1186/s12939-014-0083-5, PMID: 25252790 PMC4188876

[106] Zaborskis A Lagunaite R Busha R Lubiene J. Trend in eating habits among Lithuanian school-aged children in context of social inequality: three cross-sectional surveys 2002, 2006 and 2010. BMC Public Health. (2012) 12:52. doi: 10.1186/1471-2458-12-52, PMID: 22260778 PMC3292449

[107] Sweeting H Hunt K. Adolescent socioeconomic and school-based social status, smoking, and drinking. J Adolesc Health. (2015) 57:37–45. doi: 10.1016/j.jadohealth.2015.03.020, PMID: 26095407 PMC4510202

[108] Pape H Rossow I Andreas JB Norstrom T. Social class and alcohol use by youth: different drinking behaviors, different associations? J Stud Alcohol Drugs. (2018) 79:132–6. doi: 10.15288/jsad.2018.79.132 29227242

[109] Pförtner TK Moor I Rathmann K Hublet A Molcho M Kunst AE . The association between family affluence and smoking among 15-year-old adolescents in 33 European countries, Israel and Canada: the role of national wealth. Addiction. (2015) 110:162–73. doi: 10.1111/add.12741, PMID: 25220260

[110] de Buhr E Tannen A. Parental health literacy and health knowledge, behaviours and outcomes in children: a cross-sectional survey. BMC Public Health. (2020) 20:1096. doi: 10.1186/s12889-020-08881-5, PMID: 32660459 PMC7359277

[111] Nardone P Pierannunzio D Ciardullo S Lazzeri G Cappello N Spinelli A . Dietary habits among Italian adolescents and their relation to socio-demographic characteristics. Ann Ist Super. (2020) 56:504–13. doi: 10.4415/ANN_20_04_15, PMID: 33346179

[112] Esquius L Aguilar-Martínez A Bosque-Prous M González-Casals H Bach-Faig A Colillas-Malet E . Social inequalities in breakfast consumption among adolescents in Spain: the DESKcohort project. Nutrients. (2021) 13:2500. doi: 10.3390/nu13082500, PMID: 34444661 PMC8401108

[113] Falese L Federico B Kunst AE Perelman J Richter M Rimpela A . The association between socioeconomic position and vigorous physical activity among adolescents: a cross-sectional study in six European cities. BMC Public Health. (2021) 21:866. doi: 10.1186/s12889-021-10791-z, PMID: 33952232 PMC8097935

[114] Morgan K Lowthian E Hawkins J Hallingberg B Alhumud M Roberts C . Sugar-sweetened beverage consumption from 1998-2017: findings from the health behaviour in school-aged children/school health research network in Wales. PLoS One. (2021) 16:e0248847. doi: 10.1371/journal.pone.0248847, PMID: 33852585 PMC8046241

[115] Pedroni C Dujeu M Lebacq T Desnouck V Holmberg E Castetbon K. Alcohol consumption in early adolescence: associations with sociodemographic and psychosocial factors according to gender. PLoS One. (2021) 16:e0245597. doi: 10.1371/journal.pone.0245597, PMID: 33449956 PMC7810307

[116] Sinai T Axelrod R Shimony T Boaz M Kaufman-Shriqui V. Dietary patterns among adolescents are associated with growth, socioeconomic features, and health-related behaviors. Foods. (2021) 10:3054. doi: 10.3390/foods10123054, PMID: 34945606 PMC8700870

[117] Yang HM. Associations of socioeconomic status, parenting style, and grit with health behaviors in children using data from the panel study on Korean children (PSKC). Child Health Nurs Res. (2021) 27:309–16. doi: 10.4094/chnr.2021.27.4.309, PMID: 35004519 PMC8650947

[118] Zaborskis A Grincaitė M Kavaliauskienė A Tesler R. Family structure and affluence in adolescent eating behaviour: a cross-national study in forty-one countries. Public Health Nutr. (2021) 24:2521. doi: 10.1017/S1368980020003584 33106205 PMC10195424

[119] Lantz PM House JS Lepkowski JM Williams DR Mero RP Chen J. Socioeconomic factors, health behaviors, and mortality: results from a nationally representative prospective study of US adults. JAMA. (1998) 279:1703. doi: 10.1001/jama.279.21.1703 9624022

[120] McMaughan DJ Oloruntoba O Smith ML. Socioeconomic status and access to healthcare: interrelated drivers for healthy aging. Front Public Health. (2020) 8:231. doi: 10.3389/fpubh.2020.00231, PMID: 32626678 PMC7314918

[121] Pampel FC Krueger PM Denney JT. Socioeconomic disparities in health behaviors. Annu Rev Sociol. (2010) 36:349–70. doi: 10.1146/annurev.soc.012809.102529, PMID: 21909182 PMC3169799

[122] Fujiwara T Isumi A Ochi M. Pathway of the association between child poverty and low self-esteem: results from a population-based study of adolescents in Japan. Front Psychol. (2019) 10:937. doi: 10.3389/fpsyg.2019.00937, PMID: 31133920 PMC6511812

[123] Svendsen MT Bak CK Sørensen K Pelikan J Riddersholm SJ Skals RK . Associations of health literacy with socioeconomic position, health risk behavior, and health status: a large national population-based survey among Danish adults. BMC Public Health. (2020) 20:565. doi: 10.1186/s12889-020-08498-8, PMID: 32345275 PMC7187482

[124] Obita G Alkhatib A. Effectiveness of lifestyle nutrition and physical activity interventions for childhood obesity and associated comorbidities among children from minority ethnic groups: a systematic review and meta-analysis. Nutrients. (2023) 15:2524. doi: 10.3390/nu15112524, PMID: 37299488 PMC10255126

[125] Kuntz B Lampert T. Social disparities in parental smoking and young children’s exposure to secondhand smoke at home: a time-trend analysis of repeated cross-sectional data from the German KiGGS study between 2003-2006 and 2009-2012. BMC Public Health. (2016) 16:485. doi: 10.1186/s12889-016-3175-x, PMID: 27277721 PMC4898452

[126] Tyas SL Pederson LL. Psychosocial factors related to adolescent smoking: a critical review of the literature. Tob Control. (1998) 7:409–20. doi: 10.1136/tc.7.4.409, PMID: 10093176 PMC1751465

[127] Hiscock R Bauld L Amos A Fidler JA Munafò M. Socioeconomic status and smoking: a review. Ann N Y Acad Sci. (2012) 1248:107–23. doi: 10.1111/j.1749-6632.2011.06202.x 22092035

[128] Humensky JL. Are adolescents with high socioeconomic status more likely to engage in alcohol and illicit drug use in early adulthood? Subst Abuse Treat Prev Policy. (2010) 5:19. doi: 10.1186/1747-597X-5-19, PMID: 20687935 PMC2924306

[129] Torchyan AA Houkes I Bosma H. Income inequality and socioeconomic disparities in alcohol use among eastern European adolescents: a multilevel analysis. J Adolesc Health. (2023) 73:347–51. doi: 10.1016/j.jadohealth.2023.03.001, PMID: 37125987

[130] Kuppens S Moore SC Gross V Lowthian E Siddaway AP. The enduring effects of parental alcohol, tobacco, and drug use on child well-being: a multilevel meta-analysis. Dev Psychopathol. (2020) 32:765. doi: 10.1017/S0954579419000749 31274064 PMC7525110

[131] Patrick ME Wightman P Schoeni RF Schulenberg JE. Socioeconomic status and substance use among young adults: a comparison across constructs and drugs. J Stud Alcohol Drugs. (2012) 73:772. doi: 10.15288/jsad.2012.73.772 22846241 PMC3410945

[132] Donovan JE Molina BS. Children’s introduction to alcohol use: sips and tastes. Clin Exp Res. (2008) 32:108–19. doi: 10.1111/j.1530-0277.2007.00565.x, PMID: 18070249 PMC2212613

[133] Skylstad V Babirye JN Kiguli J Skar AMS Kühl MJ Nalugya JS . Are we overlooking alcohol use by younger children? BMJ Paediatr Open. (2022) 6:e001242. doi: 10.1136/bmjpo-2021-001242, PMID: 36053657 PMC8905875

[134] Wijtzes AI Jansen W Bouthoorn SH Pot N Hofman A Jaddoe VW . Social inequalities in young children’s sports participation and outdoor play. Int J Behav Nutr Phys Act. (2014) 11:155. doi: 10.1186/s12966-014-0155-3, PMID: 25510552 PMC4272790

[135] Gonzalo-Almorox E Urbanos-Garrido RM. Decomposing socio-economic inequalities in leisure-time physical inactivity: the case of Spanish children. Int J Equity Health. (2016) 15:106. doi: 10.1186/s12939-016-0394-9 27406235 PMC4942941

[136] Story M Neumark-Sztainer D French S. Individual and environmental influences on adolescent eating behaviors. J Am Diet Assoc. (2002) 102:S40–51. doi: 10.1016/S0002-8223(02)90421-9 11902388

[137] Federico B Falese L Capelli G. Socio-economic inequalities in physical activity practice among Italian children and adolescents: a cross-sectional study. J Public Health. (2009) 17:377–84. doi: 10.1007/s10389-009-0267-4, PMID: 21088692 PMC2967259

[138] Lampinen EK Eloranta AM Haapala EA Lindi V Väistö J Lintu N . Physical activity, sedentary behaviour, and socioeconomic status among Finnish girls and boys aged 6–8 years. Eur J Sport Sci. (2017) 17:462. doi: 10.1080/17461391.2017.1294619 28276910

[139] Musić Milanović S Buoncristiano M Križan H Rathmes G Williams J Hyska J . Socioeconomic disparities in physical activity, sedentary behavior and sleep patterns among 6-to 9-year-old children from 24 countries in the WHO European region. Obes Rev. (2021) 22:e13209. doi: 10.1111/obr.13209 34235843

[140] Moore LV Roux AVD Evenson KR McGinn AP Brines SJ. Availability of recreational resources in minority and low socioeconomic status areas. Am J Prev Med. (2008) 34:16–22. doi: 10.1016/j.amepre.2007.09.021, PMID: 18083446 PMC2254179

[141] Hoffimann E Barros H Ribeiro AI. Socioeconomic inequalities in green space quality and accessibility—evidence from a southern European city. Int J Environ Res Public Health. (2017) 14:916. doi: 10.3390/ijerph14080916, PMID: 28809798 PMC5580619

[142] Agrawal S Kim R Gausman J Sharma S Sankar R Joe W . Socio-economic patterning of food consumption and dietary diversity among Indian children: evidence from NFHS-4. Eur J Clin Nutr. (2019) 73:1361–72. doi: 10.1038/s41430-019-0406-0, PMID: 30809007

[143] Leyva RPP Mengelkoch S Gassen J Ellis BJ Russell EM Hill SE. Low socioeconomic status and eating in the absence of hunger in children aged 3–14. Appetite. (2020) 154:104755. doi: 10.1016/j.appet.2020.104755, PMID: 32579973

[144] Shinwell J Defeyter MA. Food insecurity: a constant factor in the lives of low-income families in Scotland and England. Front Public Health. (2021) 9:588254. doi: 10.3389/fpubh.2021.588254, PMID: 34095040 PMC8170021

[145] Attorp A Scott JE Yew AC Rhodes RE Barr SI Naylor PJ. Associations between socioeconomic, parental and home environment factors and fruit and vegetable consumption of children in grades five and six in British Columbia, Canada. BMC Public Health. (2014) 14:150. doi: 10.1186/1471-2458-14-150 24517088 PMC3929144

[146] Zarnowiecki D Ball K Parletta N Dollman J. Describing socioeconomic gradients in children’s diets–does the socioeconomic indicator used matter? Int J Behav Nutr Phys Act. (2014) 11:44. doi: 10.1186/1479-5868-11-44 24674231 PMC3986827

[147] Vilela S Oliveira A Pinto E Moreira P Barros H Lopes C. The influence of socioeconomic factors and family context on energy-dense food consumption among 2-year-old children. Eur J Clin Nutr. (2015) 69:47–54. doi: 10.1038/ejcn.2014.140, PMID: 25052226

[148] Janicijevic KM Kocic SS Radevic SR Jovanovic MR Radovanovic SM. Socioeconomic factors associated with psychoactive substance abuse by adolescents in Serbia. Front Pharmacol. (2017) 8:366. doi: 10.3389/fphar.2017.00366, PMID: 28659800 PMC5468426

