# Supplementary material for: Socioeconomic status and health behavior in children and adolescents: a systematic literature review
Source: Front Public Health. 2023 Oct 17;11:1228632. doi: 10.3389/fpubh.2023.1228632 (PMC10616829; doi:10.3389/fpubh.2023.1228632)
Supplement: Supplementary file 1 [file Data_Sheet_1.doc]

**Supplementary Material**

**Table S1: Search strategy**

| **S.N.** | **Database** | **Search Strategy** | **Field search** | **Limits/filters** | **Number of results** | **Remarks** |
| --- | --- | --- | --- | --- | --- | --- |
| #1 | Web of Science | (socioeconomic OR “socio-economic”) AND (“health behaviour” OR “health behavior” OR “health behaviours” OR “health behaviors”) AND (teen* OR adolescen* OR child* OR “young person” OR “young people”) | ((TS= (Socioeconomic OR “socio-economic”)) AND TS=(“health behaviour” OR “health behavior” OR “health behaviours” OR “health behaviors”)) AND TS=(teen* OR adolescen* OR child*) | Publication year 2000 to January 2022; publication type=articles, review articles, early access | 1716 |  |
| #2 | EBSCOHost | (socioeconomic OR “socio-economic”) AND (“health behaviour” OR “health behavior” OR “health behaviours” OR “health behaviors”) AND (teen* OR adolescen* OR child* OR “young person” OR “young people”) | TI (Socioeconomic OR “socio-economic”) AND AB (“health behaviour” OR “health behavior” OR “health behaviours” OR " health behaviors") AND SU (teen* OR adolescen* OR child* ) | Publication year 2000 to January 2022; publication type=articles, review articles, early access | 185 | Academic Search Ultimate, CINAHL with Full Text, Health Source: Nursing/Academic Edition, Psychology and Behavioral Sciences Collection, APA PsycArticles, APA PsycInfo, Sociology Source Ultimate |
| #3 | ScienceDirect | (socioeconomic OR “socio-economic”) AND (“health behaviour” OR “health behavior” OR “health behaviours” OR “health behaviors”) AND (teen* OR adolescen* OR child* OR “young person” OR “young people”) | Title, abstract, keywords: (Socioeconomic OR “socio-economic”) AND (“health behaviour” OR “health behavior” OR “health behaviours”) AND (teenager OR adolescent OR child) | Publication year 2000 to January 2022; publication type=articles, review articles, early access | 143 |  |
| #4 | PubMed | (Socioeconomic OR “socio-economic”) AND (“health behaviour” OR “health behavior” OR “health behaviours” OR “health behaviors”) AND (teen* OR adolescen* OR child*) | ((Socioeconomic [Title/Abstract] OR "socio-economic"[Title/Abstract]) AND ("health behaviour"[Title/Abstract] OR "health behavior"[Title/Abstract] OR "health behaviours"[Title/Abstract])) AND (teen*[Other Term] OR adolescen*[Other Term] OR child*[Other Term]) | Publication year 2000 to January 2022; publication type=articles, review articles, early access | 347 |  |
| **Total** | **Search #1+** |  | **Search#2+search#3+** | **Search#4** | **2391** |  |

**Table S2: Template for building a search strategy**

| Key concept #1 |  | Key concept #2 | Key concept #3 | Possible additional keywords | Date range | Limiters? |
| --- | --- | --- | --- | --- | --- | --- |
| socioeconomic  OR  “socio-economic” |  | AND  AND  “health behaviour”  OR  “health behavior”  OR  “health behaviours”  OR  “health behaviors” | teen*  OR  adolescen*  OR  child*  OR  “young person”  OR  “young people” | **status**  **indicators** | 2000 onwards | **Language:**  English  **Type of resource**:  journal articles (peer-reviewed) |

**Table S3: PRISMA 2020 Checklist**

| **Section/topic** | **#** | **Checklist item** | **Reported on page #** |
| --- | --- | --- | --- |
| **TITLE** | | |  |
| Title | 1 | Identify the report as a systematic review, meta-analysis, or both. |  |
| **ABSTRACT** | | |  |
| Structured summary | 2 | Provide a structured summary including, as applicable: background; objectives; data sources; study eligibility criteria, participants, and interventions; study appraisal and synthesis methods; results; limitations; conclusions and implications of key findings; systematic review registration number. |  |
| **INTRODUCTION** | | |  |
| Rationale | 3 | Describe the rationale for the review in the context of what is already known. |  |
| Objectives | 4 | Provide an explicit statement of questions being addressed with reference to participants, interventions, comparisons, outcomes, and study design (PICOS). |  |
| **METHODS** | | |  |
| Protocol | 5 | Indicate if a review protocol exists, if and where it can be accessed (e.g., Web address), and, if available, provide registration information including registration number. |  |
| Eligibility criteria | 6 | Specify study characteristics (e.g., PICOS, length of follow-up) and report characteristics (e.g., years considered, language, publication status) used as criteria for eligibility, giving rationale. |  |
| Information sources | 7 | Describe all information sources (e.g., databases with dates of coverage, contact with study authors to identify additional studies) in the search and date last searched. |  |
| Search | 8 | Present full electronic search strategy for at least one database, including any limits used, such that it could be repeated. |  |
| Study selection | 9 | State the process for selecting studies (i.e., screening, eligibility, included in systematic review, and, if applicable, included in the meta-analysis). |  |
| Data collection process | 10 | Describe method of data extraction from reports (e.g., piloted forms, independently, in duplicate) and any processes for obtaining and confirming data from investigators. |  |
| Data items | 11 | List and define all variables for which data were sought (e.g., PICOS, funding sources) and any assumptions and simplifications made. |  |
| Risk of bias in individual studies | 12 | Describe methods used for assessing risk of bias of individual studies (including specification of whether this was done at the study or outcome level), and how this information is to be used in any data synthesis. |  |
| Summary measures | 13 | State the principal summary measures (e.g., risk ratio, difference in means). |  |
| Synthesis of results | 14 | Describe the methods of handling data and combining results of studies, if done, including measures of consistency for each review or meta-analysis. |  |
| Risk of bias across studies | 15 | Specify any assessment of risk of bias that may affect the cumulative evidence (e.g., publication bias, selective reporting within studies). |  |
| Additional analyses | 16 | Describe methods of additional analyses (e.g., sensitivity or subgroup analyses, meta-regression), if done, indicating which were pre-specified. |  |

| **RESULTS** | | |  |
| --- | --- | --- | --- |
| Study selection | 17 | Give numbers of studies screened, assessed for eligibility, and included in the review, with reasons for exclusions at each stage, ideally with a flow diagram. |  |
| Study characteristics | 18 | For each study, present characteristics for which data were extracted (e.g., study size, PICOS, follow-up period) and provide the citations. |  |
| Risk of bias within studies | 19 | Present data on risk of bias of each study and, if available, any outcome level assessment (see item 12). |  |
| Results of individual studies | 20 | For all outcomes considered (benefits or harms), present, for each study: (a) simple summary data for each intervention group (b) effect estimates and confidence intervals, ideally with a forest plot. |  |
| Synthesis of results | 21 | Present results of each systematic review or meta-analysis done, including confidence intervals and measures of consistency. |  |
| Risk of bias across studies | 22 | Present results of any assessment of risk of bias across studies (see Item 15). |  |
| Additional analysis | 23 | Give results of additional analyses, if done (e.g., sensitivity or subgroup analyses, meta-regression [see Item 16]). |  |
| **DISCUSSION** | | |  |
| Summary of evidence | 24 | Summarize the main findings including the strength of evidence for each main outcome; consider their relevance to key groups (e.g., healthcare providers, users, and policy makers). |  |
| Limitations | 25 | Discuss limitations at study and outcome level (e.g., risk of bias), and at review-level (e.g., incomplete retrieval of identified research, reporting bias). |  |
| Conclusions | 26 | Provide a general interpretation of the results in the context of other evidence, and implications for future research. |  |
| **FUNDING** | | |  |
| Funding | 27 | Describe sources of funding for the systematic review and other support (e.g., supply of data); role of funders for the systematic review. |  |

#### **Table S4: Critical Appraisal Results Using JBI SUMARI**

| **Citation** | **Q1** | **Q2** | **Q3** | **Q4** | **Q5** | **Q6** | **Q7** | **Q8** |
| --- | --- | --- | --- | --- | --- | --- | --- | --- |
| Al Sabbah et al 2007. | Y | Y | Y | Y | U | U | Y | Y |
| Andersen et al 2007. | Y | Y | Y | Y | Y | Y | Y | Y |
| de Buhr et al. 2020. | Y | Y | Y | Y | Y | Y | Y | Y |
| Doku et al 2010. | Y | Y | Y | Y | U | U | Y | Y |
| Doku et al 2012. | Y | Y | Y | Y | U | Y | Y | Y |
| Esquius et al. 2021. | Y | Y | Y | Y | Y | Y | Y | Y |
| Falese et al. 2021. | Y | Y | Y | Y | Y | Y | Y | Y |
| Hankonen et al. 2017. | Y | Y | Y | Y | Y | Y | Y | Y |
| Henriksen et al 2016. | Y | Y | Y | Y | U | U | Y | Y |
| Johansen et al 2006. | Y | Y | Y | Y | U | U | Y | Y |
| Kislitsyna et al 2010. | Y | Y | Y | Y | Y | U | Y | Y |
| Krist et al. 2017. | Y | Y | Y | Y | Y | Y | Y | Y |
| Lazzeri et al 2014. | Y | Y | Y | Y | U | U | Y | Y |
| Levin et al 2014. | Y | Y | Y | Y | U | U | Y | Y |
| Liu et al 2013. | Y | Y | Y | Y | Y | Y | Y | Y |
| Liu et al 2016. | Y | Y | Y | Y | Y | Y | Y | Y |
| Melotti et al 2011. | Y | Y | Y | Y | Y | Y | Y | Y |
| Mikki et al 2010. | Y | Y | Y | Y | Y | Y | Y | Y |
| Moor et al. 2015. | Y | Y | Y | Y | Y | Y | Y | Y |
| Moore et al 2015. | Y | Y | Y | Y | Y | Y | Y | Y |
| Nardone et al. 2020. | Y | Y | Y | Y | U | U | Y | Y |
| Pape et al 2018. | Y | Y | Y | Y | U | U | Y | Y |
| Park et al 2017. | Y | Y | Y | Y | Y | Y | Y | Y |
| Pavon et al. 2010. | Y | Y | Y | Y | N/A | N/A | Y | Y |
| Pedroni et al 2021. | Y | Y | Y | Y | Y | Y | Y | Y |
| Pförtner et al. 2015. | Y | Y | Y | Y | Y | Y | Y | Y |
| Richter et al 2009. | Y | Y | Y | Y | Y | Y | Y | Y |
| Richter 2006. | Y | Y | Y | Y | Y | Y | Y | Y |
| Richter et al 2009. | Y | Y | Y | Y | Y | Y | Y | Y |
| Simetin et al 2013. | Y | Y | Y | Y | Y | Y | Y | Y |
| Simetin et al 2011. | Y | Y | Y | Y | U | U | Y | Y |
| Sinai et al 2021. | Y | Y | Y | Y | Y | Y | Y | Y |
| Svastisalee et al 2012. | Y | Y | Y | Y | Y | Y | Y | Y |
| Sweeting, & Hunt 2015. | Y | Y | Y | Y | Y | Y | Y | Y |
| Vereecken et al. 2005. | Y | Y | Y | Y | U | U | Y | Y |
| Voráčová et al 2016. | Y | Y | Y | Y | Y | Y | Y | Y |
| Yannakoulia et al. 2016. | Y | Y | Y | Y | Y | Y | Y | Y |
| Zaborskis et al 2021. | Y | Y | Y | Y | Y | U | Y | Y |
| Zaborskis et al 2012. | Y | Y | Y | Y | Y | Y | Y | Y |
| **%** | **100.0** | **100.0** | **100.0** | **100.0** | **69.23** | **66.66** | **100.0** | **100.0** |

**Table S4: Cohort Study**

| **Citation** | **Q1** | **Q2** | **Q3** | **Q4** | **Q5** | **Q6** | **Q7** | **Q8** | **Q9** | **Q10** | **Q11** |
| --- | --- | --- | --- | --- | --- | --- | --- | --- | --- | --- | --- |
| Andersen et al 2008. | Y | Y | Y | Y | Y | Y | Y | Y | Y | Y | Y |
| Doku et al 2010. | Y | Y | Y | Y | U | Y | Y | Y | Y | N/A | Y |
| et al 2018. | Y | N | Y | Y | Y | Y | Y | N | Y | Y | Y |
| Melotti et al 2013. | Y | Y | Y | Y | Y | Y | Y | Y | N/A | Y | Y |
| Morgan et al. 2021. | Y | Y | Y | Y | Y | Y | Y | Y | N/A | Y | Y |
| Poulain T et al 2019. | Y | Y | Y | U | Y | Y | Y | Y | Y | N | Y |
| Yang. 2021. | Y | Y | Y | Y | U | Y | Y | N/A | Y | Y | Y |
| **%** | **100.0** | **85.71** | **100.0** | **85.711** | **71.42** | **100.0** | **100.0** | **71.42** | **71.42** | **71.42** | **100.0** |

**Note: Y= Yes, N=No, U= Unknown, N/A= Not available**

|  | **Table S5: Risk of Bias assessment Tool of Eligible Articles by using the Hoy 2012 tool** | | | | | | | | | | |  |
| --- | --- | --- | --- | --- | --- | --- | --- | --- | --- | --- | --- | --- |
| NO | **Study ID** | **Representation** | **Sampling** | **Random selection** | **Non response bias** | **Data collection** | **Case Definition** | **Reliability and validity of study tool** | **Method of data collection** | **Prevalence period** | **Numerator and denominator** | **Summary Assessment** |
| 1 | Al Sabbah et al. | High risk | Low risk | High risk | Low risk | Low risk | High risk | High risk | High risk | low risk | Low risk | High risk |
| 2 | Andersen et al. | High risk | Low risk | High risk | Low risk | Low risk | High risk | High risk | High risk | low risk | Low risk | High risk |
| 3 | Andersen et al. | High risk | Low risk | Low risk | High risk | Low risk | High risk | Low risk | Low risk | Low risk | Low risk | Medium risk |
| 4 | de Buhr et al. | Low risk | Low risk | Low risk | Low risk | Low risk | High risk | Low risk | Low risk | Low risk | Low risk | Low risk |
| 5 | Doku et al. | Low risk | Low risk | Low risk | Low risk | Low risk | High risk | Low risk | Low risk | Low risk | Low risk | Low risk |
| 6 | Doku et al. | Low risk | Low risk | High risk | Low risk | High risk | High risk | High risk | Low risk | Low risk | Low risk | High risk |
| 7 | Doku et al. | High risk | Low risk | Low risk | High risk | Low risk | High risk | Low risk | Low risk | Low risk | Low risk | Medium risk |
| 8 | Esquius et al. | High risk | Low risk | Low risk | Low risk | Low risk | Low risk | Low risk | Low risk | Low risk | Low risk | Low risk |
| 9 | Falese et al. | High risk | Low risk | High risk | Low risk | High risk | Low risk | High risk | Low risk | Low risk | Low risk | Medium risk |
| 10 | Hankonen et al. | High risk | Low risk | Low risk | Low risk | Low risk | Low risk | Low risk | Low risk | Low risk | Low risk | Low risk |
| 11 | Henriksen et al. | High risk | Low risk | Low risk | Low risk | Low risk | Low risk | Low risk | Low risk | Low risk | Low risk | Low risk |
| 12 | Johansen et al. | High risk | High risk | Low risk | Low risk | Low risk | Low risk | Low risk | Low risk | Low risk | Low risk | Low risk |
| 13 | Kislitsyna et al. | Low risk | Low risk | Low risk | Low risk | Low risk | Low risk | Low risk | Low risk | Low risk | Low risk | Low risk |
| 14 | Krist et al. | High risk | Low risk | Low risk | Low risk | Low risk | High risk | Low risk | Low risk | Low risk | Low risk | Low risk |
| 15 | Lazzeri et al. | High risk | Low risk | Low risk | High risk | High risk | High risk | High risk | Low risk | High risk | Low risk | High risk |
| 16 | Lee et al. | High risk | Low risk | Low risk | High risk | Low risk | High risk | Low risk | Low risk | Low risk | Low risk | Medium risk |
| 17 | Levin et al. | High risk | Low risk | Low risk | High risk | Low risk | Low risk | Low risk | Low risk | Low risk | Low risk | Low risk |
| 18 | Liu et al. | Low risk | Low risk | Low risk | Low risk | Low risk | Low risk | Low risk | Low risk | Low risk | Low risk | Low risk |
| 19 | Liu et al. | Low risk | Low risk | Low risk | Low risk | Low risk | High risk | Low risk | Low risk | Low risk | Low risk | Low risk |
| 20 | Melotti et al. | Low risk | Low risk | Low risk | Low risk | Low risk | Low risk | Low risk | Low risk | Low risk | Low risk | Low risk |
| 21 | Melotti et al. | High risk | Low risk | Low risk | High risk | Low risk | High risk | Low risk | Low risk | Low risk | Low risk | Medium risk |
| 22 | Mikki et al. | Low risk | Low risk | Low risk | Low risk | Low risk | Low risk | Low risk | Low risk | Low risk | Low risk | Low risk |
| 23 | Moor et al. | High risk | Low risk | Low risk | High risk | Low risk | High risk | Low risk | Low risk | Low risk | Low risk | Medium risk |
| 24 | Moore et al. | High risk | Low risk | Low risk | Low risk | Low risk | High risk | Low risk | Low risk | Low risk | Low risk | Low risk |
| 25 | Morgan et al. | High risk | Low risk | Low risk | High risk | Low risk | High risk | Low risk | Low risk | Low risk | Low risk | Medium risk |
| 26 | Nardone et al. | High risk | Low risk | Low risk | High risk | High risk | High risk | High risk | Low risk | High risk | Low risk | High risk |
| 27 | Pape et al. | High risk | Low risk | Low risk | High risk | Low risk | Low risk | Low risk | Low risk | Low risk | Low risk | Low risk |
| 28 | Park et al. | Low risk | Low risk | Low risk | Low risk | Low risk | Low risk | Low risk | Low risk | Low risk | Low risk | Low risk |
| 29 | Pavon et al. | Low risk | Low risk | Low risk | Low risk | Low risk | High risk | Low risk | Low risk | Low risk | Low risk | Low risk |
| 30 | Pedroni et al. | Low risk | Low risk | Low risk | Low risk | Low risk | Low risk | Low risk | Low risk | Low risk | Low risk | Low risk |
| 31 | Pförtner et al. | Low risk | Low risk | Low risk | Low risk | Low risk | Low risk | Low risk | Low risk | Low risk | Low risk | Low risk |
| 32 | Poulain et al. | High risk | Low risk | Low risk | High risk | Low risk | High risk | Low risk | Low risk | Low risk | Low risk | Medium risk |
| 33 | Richter et al. | High risk | Low risk | Low risk | High risk | Low risk | High risk | Low risk | Low risk | Low risk | Low risk | Medium risk |
| 34 | Richter et al. | High risk | Low risk | Low risk | Low risk | Low risk | High risk | Low risk | Low risk | Low risk | Low risk | Low risk |
| 35 | Richter et al. | High risk | Low risk | Low risk | High risk | High risk | High risk | High risk | Low risk | High risk | Low risk | High risk |
| 36 | Simetin et al. | High risk | Low risk | Low risk | High risk | Low risk | Low risk | Low risk | Low risk | Low risk | Low risk | Low risk |
| 37 | Simetin et al. | Low risk | Low risk | Low risk | Low risk | Low risk | Low risk | Low risk | Low risk | Low risk | Low risk | Low risk |
| 38 | Sinai et al. | Low risk | Low risk | Low risk | Low risk | Low risk | High risk | Low risk | Low risk | Low risk | Low risk | Low risk |
| 39 | Svastisalee et al. | Low risk | Low risk | Low risk | Low risk | Low risk | Low risk | Low risk | Low risk | Low risk | Low risk | Low risk |
| 40 | Sweeting et al. | Low risk | Low risk | Low risk | Low risk | Low risk | Low risk | Low risk | Low risk | Low risk | Low risk | Low risk |
| 41 | Vereecken et al. | High risk | Low risk | Low risk | High risk | Low risk | High risk | Low risk | Low risk | Low risk | Low risk | Medium risk |
| 42 | Voráčová et al. | High risk | Low risk | Low risk | Low risk | Low risk | High risk | Low risk | Low risk | Low risk | Low risk | Low risk |
| 43 | Yannakoulia et al. | High risk | Low risk | Low risk | High risk | High risk | High risk | High risk | Low risk | High risk | Low risk | High risk |
| 44 | Yang et al. | High risk | Low risk | Low risk | High risk | Low risk | Low risk | Low risk | Low risk | Low risk | Low risk | Low risk |
| 45 | Zaborskis et al. | High risk | Low risk | Low risk | High risk | Low risk | Low risk | Low risk | Low risk | Low risk | Low risk | Low risk |
| 46 | Zaborskis et al. | Low risk | Low risk | Low risk | Low risk | Low risk | Low risk | Low risk | Low risk | Low risk | Low risk | Low risk |
|  |  |  |  |  |  |  |  |  |  |  |  |  |
|  |  |  |  |  |  |  |  |  |  |  |  |  |
|  |  |  |  |  |  |  |  |  |  |  |  |  |
|  | **Risk of bias assessment tool: Yes (low risk); No (high risk)** | | | | | | | | | | |  |
|  | 1. Representation: Was the study population a close representation of the national population? | | | | | | | | | | |  |
|  | 2. Sampling: Was the sampling frame a true or close representation of the target population? | | | | | | | | | | |  |
|  | 3. Random selection: Was some form of random selection used to select the sample OR was a census undertaken? | | | | | | | | | | |  |
|  | 4. Non-response bias: Was the likelihood of non-response bias minimal? | | | | | | | | | | |  |
|  | 5. Data collection: Were data collected directly from the subjects? | | | | | | | | | | |  |
|  | 6. Case definition: Was an acceptable case definition used in the study? | | | | | | | | | | |  |
|  | 7. Reliability and validity of study tool: Was the study instrument that measured the parameter of interest show to have reliability and validity? | | | | | | | | | | |  |
|  | 8. Data collection: Was the same mode of data collection used for all subjects? | | | | | | | | | | |  |
|  | 9. Prevalence period: Was the length of the prevalence period for the parameter of interest appropriate? | | | | | | | | | | |  |
|  | 10. Numerators and denominators: Were the numerator(s) and denominator(s) for the parameter of interest appropriate?  **The overall risk of bias scored based on the number of high risk of bias per study: low risk (≤2), moderate risk (3–4), and high risk (≥5).** | | | | | | | | | | |  |
